# MLK3 mediates impact of PKG1**α** on cardiac function and controls blood pressure through separate mechanisms

**DOI:** 10.1172/jci.insight.149075

**Published:** 2021-09-22

**Authors:** Timothy D. Calamaras, Suchita Pande, Robert A.U. Baumgartner, Seung Kyum Kim, Joseph C. McCarthy, Gregory L. Martin, Kelly Tam, Angela L. McLaughlin, Guang-rong Wang, Mark J. Aronovitz, Weiyu Lin, Jonathan I. Aguirre, Paulina Baca, Peiwen Liu, Daniel A. Richards, Roger J. Davis, Richard H. Karas, Iris Z. Jaffe, Robert M. Blanton

**Affiliations:** 1 Molecular Cardiology Research Institute and; 2 Graduate School of Biomedical Sciences, Tufts University, Boston, Massachusetts, USA.; 3 University of Massachusetts School of Medicine, Worchester, Massachusetts, USA.

**Keywords:** Cardiology, Cyclic nucleotides, Heart failure, Hypertension

## Abstract

cGMP-dependent protein kinase 1α (PKG1α) promotes left ventricle (LV) compensation after pressure overload. PKG1-activating drugs improve heart failure (HF) outcomes but are limited by vasodilation-induced hypotension. Signaling molecules that mediate PKG1α cardiac therapeutic effects but do not promote PKG1α-induced hypotension could therefore represent improved therapeutic targets. We investigated roles of mixed lineage kinase 3 (MLK3) in mediating PKG1α effects on LV function after pressure overload and in regulating BP. In a transaortic constriction HF model, PKG activation with sildenafil preserved LV function in MLK3^+/+^ but not MLK3^–/–^ littermates. MLK3 coimmunoprecipitated with PKG1α. MLK3-PKG1α cointeraction decreased in failing LVs. PKG1α phosphorylated MLK3 on Thr277/Ser281 sites required for kinase activation. MLK3^–/–^ mice displayed hypertension and increased arterial stiffness, though PKG stimulation with sildenafil or the soluble guanylate cyclase (sGC) stimulator BAY41-2272 still reduced BP in MLK3^–/–^ mice. MLK3 kinase inhibition with URMC-099 did not affect BP but induced LV dysfunction in mice. These data reveal MLK3 as a PKG1α substrate mediating PKG1α preservation of LV function but not acute PKG1α BP effects. Mechanistically, MLK3 kinase–dependent effects preserved LV function, whereas MLK3 kinase–independent signaling regulated BP. These findings suggest augmenting MLK3 kinase activity could preserve LV function in HF but avoid hypotension from PKG1α activation.

## Graphical abstract

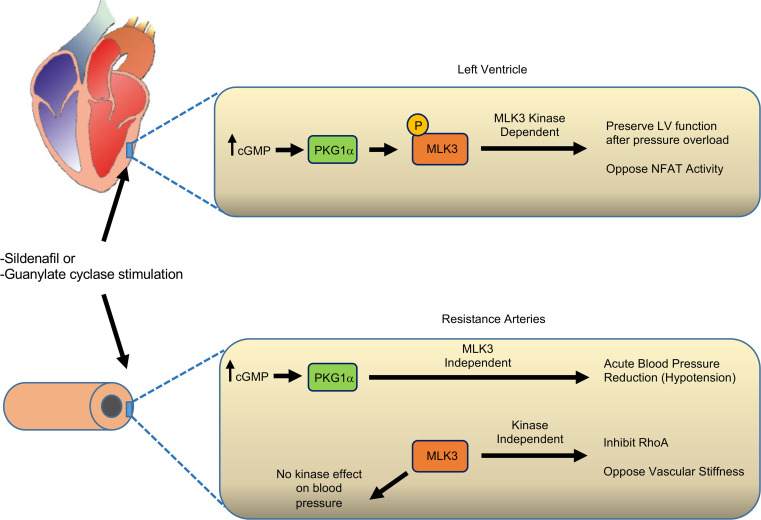

## Introduction

The cGMP-dependent protein kinase 1 (PKG1) regulates left ventricular function (1) and BP (2). Agents that activate PKG1 through augmentation of cGMP, including nitrates (3), sacubitril/valsartan (4), and vericiguat (5), now represent a rapidly emerging class of drugs that has improved outcomes in heart failure (HF) with reduced ejection fraction. However, hypotension arising from PKG1-induced vasodilation remains a major limitation of these agents (6). Signaling molecules that mediate PKG1 therapeutic effects in the left ventricle (LV) but do not promote PKG1 effects on BP could therefore represent attractive candidate therapeutic targets for HF. Currently, the specific substrates that mediate PKG1 effects on LV function and on BP remain incompletely understood.

The intracellular signaling molecule mixed lineage kinase 3 (MLK3) has recently been identified as opposing pressure overload–induced LV remodeling and dysfunction (7), but its regulation by PKG1 has not been studied. MLK3 is expressed in multiple tissues, including cardiac and vascular tissue (7, 8). MLK3 directly phosphorylates MAPKs and other substrates through kinase-dependent mechanisms (9) but also inhibits the activation of the small GTPase RhoA through allosteric, kinase-independent mechanisms (10). Like PKG1 (1), MLK3 is required for preservation of LV function early after pressure overload and promotes initial compensatory JNK activation in the cardiac myocyte and LV (7). MLK3 also regulates vascular processes including angiogenesis (11) and vascular smooth muscle injury response (8), though the effect of MLK3 on vascular contribution to BP has not been explored. Finally, MLK3 contains a leucine zipper (LZ) protein interaction domain with high homology to the LZ domain of the PKG1α isoform (12), suggesting the potential for a MLK3–PKG1α cointeraction. The PKG1α LZ domain mediates PKG1α interaction with multiple cardiovascular substrates (13–16) and when mutated leads to both LV dysfunction after pressure overload (1) and chronic hypertension (2). These collective observations raise the possibility that MLK3 mediates PKG1α initial compensatory effects on LV function and/or PKG1α effects on BP, but this remains untested.

Thus, this study tested the hypothesis that MLK3 functions as a PKG1α LZ–dependent effector in the cardiovascular system that may separate the cardiovascular benefits from the hypotensive effects of PKG activation. In a MLK3 genetic deletion model, we investigated the requirement of MLK3 for early PKG1 effects on LV function after pressure overload, and on BP regulation. We examined PKG1α interaction with and activation of MLK3 in LV tissue and in vitro.

## Results

### MLK3 is necessary for the cardiac benefits of the PKG1 activator sildenafil in HF.

**Figure 1:**
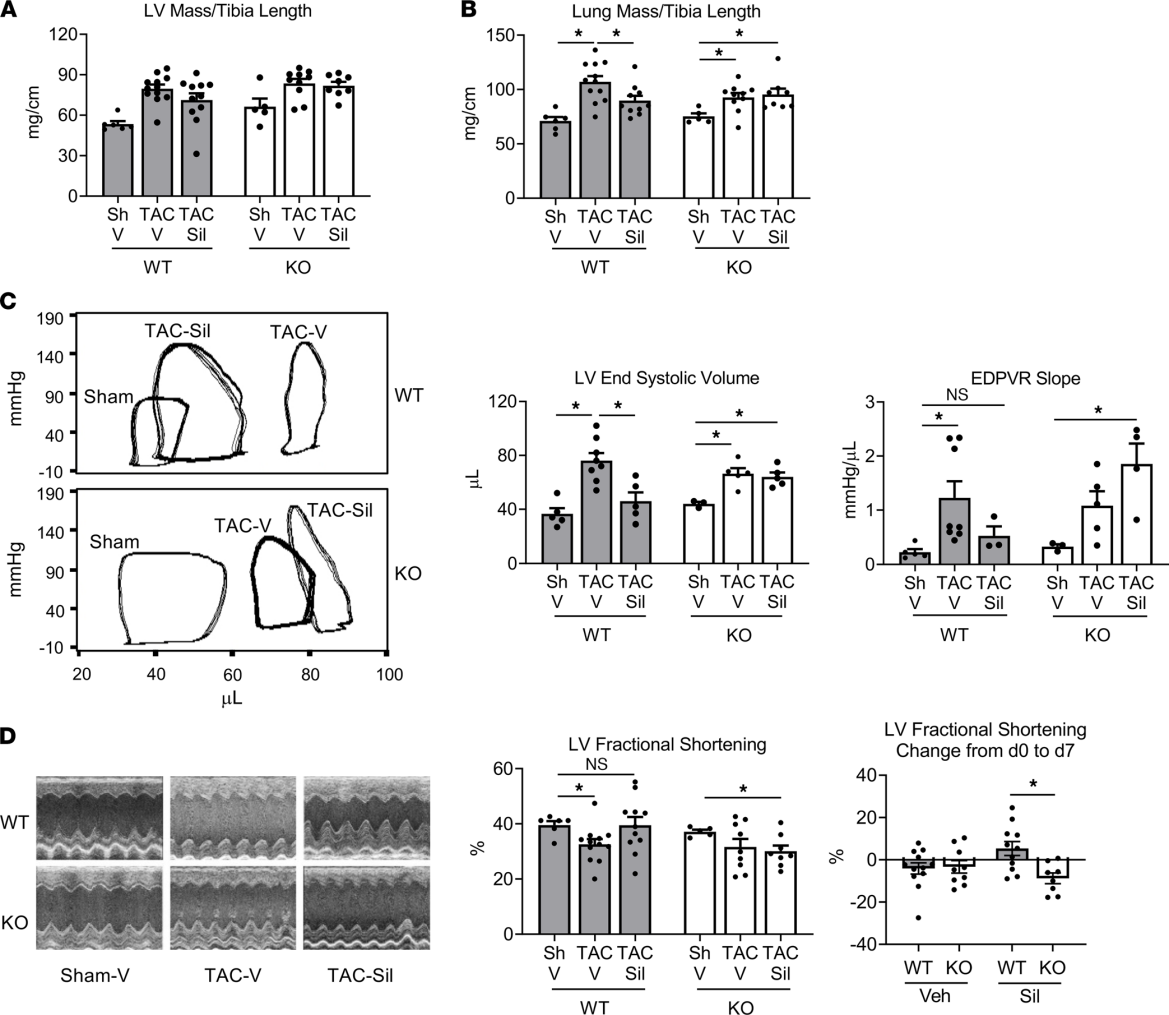
Genetic deletion of MLK3 inhibits therapeutic effects of sildenafil on LV function in pressure overload. MLK3-KO mice or WT littermates subjected to 7-day left ventricular pressure overload by TAC. (**A**) Left ventricular mass normalized to tibia length. (**B**) Pulmonary congestion assayed by lung mass normalized to tibia length. (**C**) Hemodynamic data acquired by left ventricular pressure–volume loop analysis. Representative left ventricular pressure volume loops, summary data of end systolic volume, and slope of the left ventricular EDPVR. (**D**) Representative M-mode left ventricular echocardiograms and summary data of LV fractional shortening. Change from day 0 (before TAC) to day 7 (after TAC) also shown. *P < 0.05 by Brown-Forsyth 1-way ANOVA and Welch’s multiple comparisons. *n* = 6 WT sham, 5 KO sham, 12 WT TAC V, 11 WT Sil, 9 KO TAC V, 8 MLK3-KO TAC Sil. V, vehicle; Sil, sildenafil; MLK3, mixed lineage kinase 3; LV, left ventricle; TAC, transaortic constriction; EDPVR, end diastolic pressure–volume relation.

We tested the requirement of MLK3 for the therapeutic effects of sildenafil in HF using MLK3^–/–^ mice (denoted as KO), compared with MLK3^+/+^ WT littermate controls (denoted as WT). Sildenafil inhibits phosphodiesterase 5–dependent degradation of cGMP, thereby activating PKG1α, and opposes LV dysfunction and remodeling in response to pressure overload in a PKG1α-dependent fashion (1, 17). Prior studies demonstrate a requirement for the PKG1α LZ domain for functional effects of sildenafil on LV compensation to 7 days of pressure overload (1). We therefore tested the effects of sildenafil on LV function in MLK3-KO mice using a 7-day transaortic constriction (TAC) model. Sildenafil did not significantly improve TAC-induced LV hypertrophy on day 7 (Figure 1A), consistent with the short duration of TAC. Sildenafil improved HF induced by TAC, as assayed by measuring pulmonary congestion via lung mass/tibia length, in the WT TAC mice, but did not improve pulmonary congestion in the KO TAC group (Figure 1B). Sildenafil improved end systolic volume and the slope of the end diastolic pressure volume relation in WT, but these benefits were not present in KO mice exposed to TAC (Figure 1C). Sildenafil also preserved LV fractional shortening in WT but not MLK3-KO mice (Figure 1D). Direct comparison of effects of sildenafil on LV fractional shortening between MLK3 WT and KO TAC mice demonstrated significant improvements only in the WT (Figure 1D).

**Figure 2:**
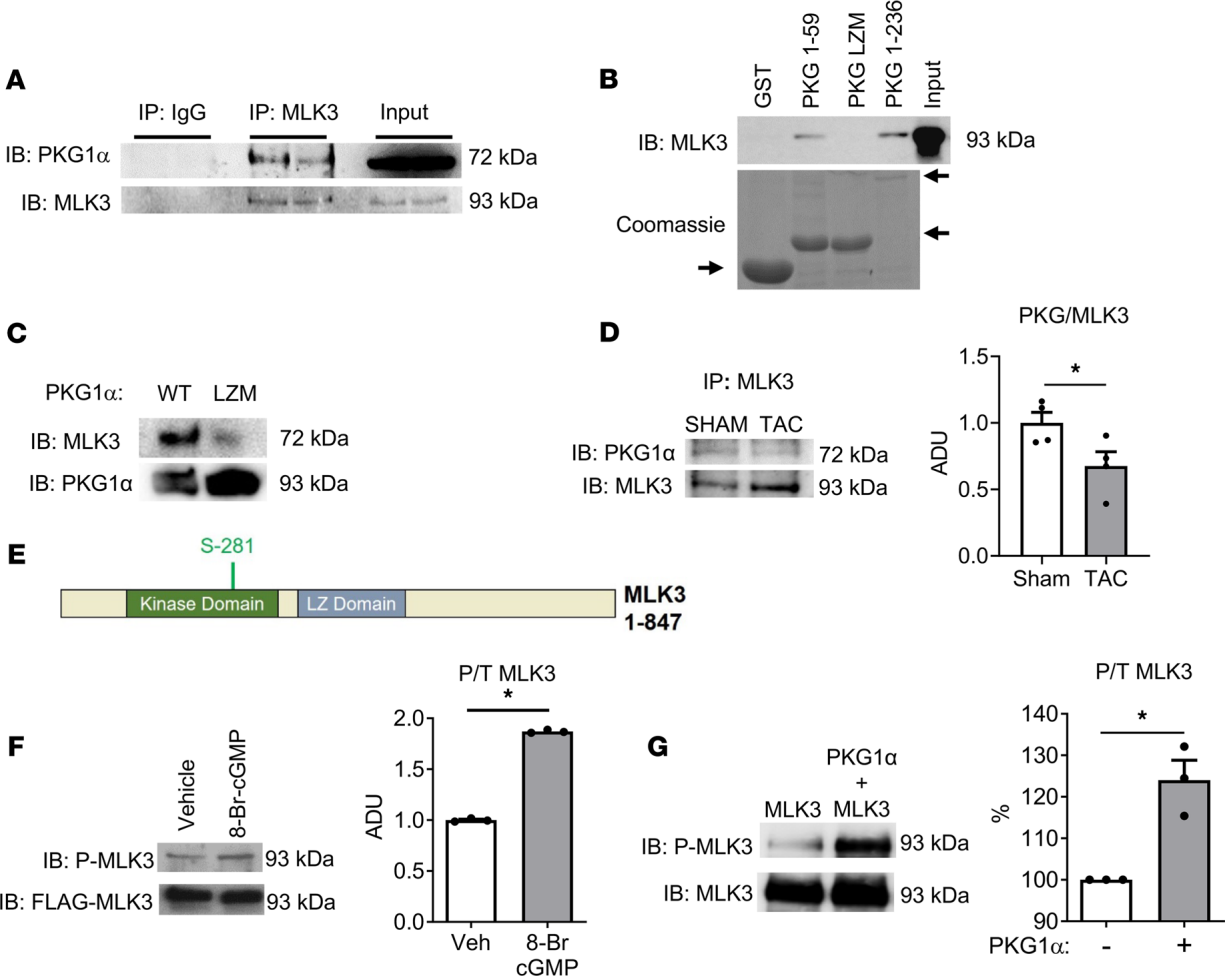
PKG1α interacts with and phosphorylates MLK3. (**A**) Coimmunoprecipitation of MLK3 and PKG1α from mouse LV lysate (representative image from *n* = 5). (**B**) Coprecipitation of the GST-tagged N-terminal PKG1α LZ domain (amino acids 1–59), and PKG1α amino acids 1 to 236, with endogenous MLK3 from mouse LV lysate. Coomassie staining confirmed protein input of GST-tagged proteins (from *n* = 3 separate experiments). Arrows denote GST fusion proteins. (**C**) Full-length PKG1α WT or LZM were affinity purified and incubated with recombinant MLK3. Coprecipitation of recombinant MLK3 detected by immunoblotting (representative of *n* = 4). (**D**) Disruption of PKG1α-MLK3 cointeraction in the pressure overloaded LV. Immunoprecipitation of MLK3 in left ventricular lysate from mice subjected to sham vs. 7-day transaortic constriction. Representative IB for PKG1α or MLK3. Graph of PKG1α normalized to MLK3 in immunoprecipitant. *n* = 4 independent experiments. *P < 0.05. (**E**) Analysis of PKG1α target sites on MLK3 using NetPhosK 1.0 reveals potential phosphorylation site Ser281 within the MLK3 kinase domain. (**F**) HEK293 cells transfected with FLAG-MLK3 were stimulated with 8-Bromo-cGMP (8-Br-cGMP, 5 mM, 30 minutes) or Veh. FLAG immunoprecipitant from lysates immunoblotted for P-MLK3 on residues Thr277/Ser281 (*n* = 3). (**G**) Phosphorylation of recombinant MLK3 on residues Thr277/Ser281 assessed in kinase reaction mixture containing recombinant MLK3 and purified PKG1α proteins (*n* = 3). For all experiments each replicate is an independent experiment. *P < 0.05 by Student’s unpaired 2-tailed *t* test. PKG1α, cGMP-dependent protein kinase 1α; MLK3, mixed lineage kinase 3; LV, left ventricle; GST, glutathione-*S*-transferase; LZ, leucine zipper; LZM, leucine zipper mutant; Veh, vehicle; ADU, arbitrary densitometric units ; P-MLK3, phosphorylation of MLK3; P/T: Ratio of phosphorylated to total; IB, immunoblot.

*PKG1αinteracts with and phosphorylates MLK3 in the LV*. To examine potential regulation of MLK3 by PKG1α, we first tested whether PKG1α and MLK3 interact in LV tissue, and we observed coimmunoprecipitation of MLK3 with PKG1α in WT mouse LV tissue lysates (Figure 2A). See complete unedited blots in the supplemental material. Conversely, glutathione-*S*-transferase (GST) pulldown studies using amino acids 1 to 236 of PKG1α precipitated MLK3 in LV tissue lysates. This interaction requires the PKG1α LZ domain, as the GST-PKG1α LZ domain was sufficient to pull down MLK3, whereas GST-PKG1α mutant LZ domain (16) or GST alone failed to precipitate MLK3 (Figure 2B). We next tested for direct interaction between purified WT or LZ mutation (LZM) full-length PKG1α incubated in vitro with recombinant MLK3. MLK3 coprecipitated with WT, but not LZM PKG1α, indicating both direct cointeraction of MLK3 and PKG1α, as well as a requirement of the PKG1α LZ domain for this interaction (Figure 2C). We examined PKG1α–MLK3 coprecipitation in the setting of pathologic LV pressure overload. We observed a 32% ± 11% reduction of PKG1α precipitation, normalized to MLK3 in LV tissue from WT mice subjected to LV pressure overload by TAC compared with sham surgery (Figure 2D).

Phosphorylation of MLK3 on threonine 277 and serine 281 induces MLK3 kinase activation (18). Bioinformatics analysis using NetPhosK software (19) revealed a potential PKG1α target site at Ser281 within the kinase domain of human MLK3 (Figure 2E). We next tested the effect of PKG1α on MLK3 phosphorylation. In HEK293 cells transfected with PKG1α and with FLAG-tagged MLK3 (20), the administration of 8-Bromo-cGMP, a membrane permeable PKG activator, induced MLK3 phosphorylation on the Thr277/Ser281 kinase activation site (Figure 2F). In vitro coincubation of purified PKG1α with MLK3 also induced MLK3 phosphorylation on these sites, supporting that MLK3 was a direct substrate of PKG1α (Figure 2G).

**Figure 3:**
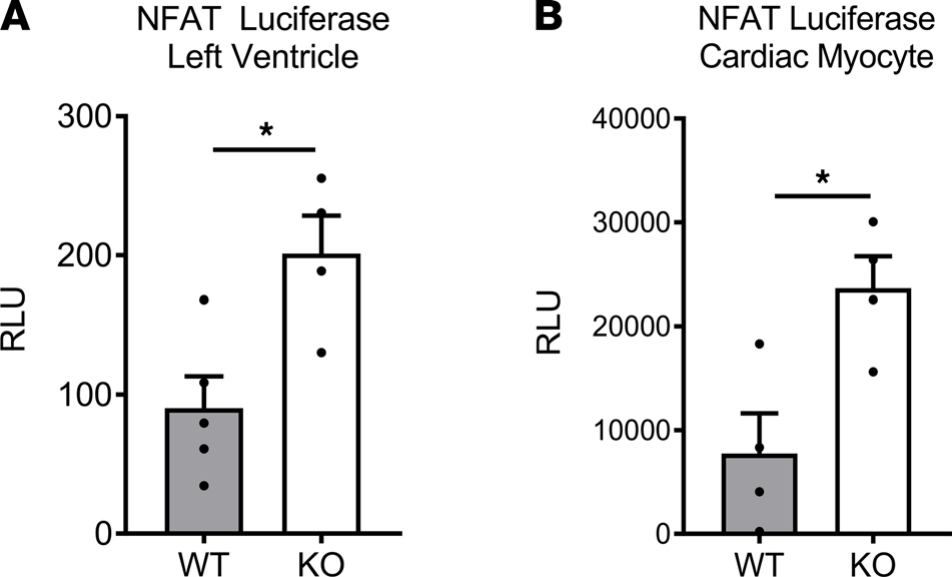
Increased cardiac myocyte NFAT activation in MLK3-KO mice. Male MLK3-KO or WT littermate controls were crossed with mice harboring transgenic expression of firefly luciferase under control of the cardiac myocyte restricted αMHC promotor and the NFAT-induced promotor (NFAT-Luc). (**A**) Luc assay of left ventricular tissue. *n* = 5 NFAT-Luc × MLK3 WT, 4 NFAT-Luc × MLK3-KO. (**B**) Luc assay from adult cardiac myocytes isolated from male NFAT-Luc × MLK3-KO mice or NFAT-Luc × MLK3 WT littermate controls. *n* = 4 per genotype. *P < 0.05 by Student’s unpaired 2-tailed *t* test. NFAT, nuclear factor of activated T cell; RLU, relative Luc units; MLK3, mixed lineage kinase 3.

To test the relevant downstream signaling mechanisms modulated by MLK3 in vivo, we examined the activation state of the prohypertrophic transcription factor nuclear factor of activated T cells (NFAT), a MLK3 kinase–dependent effector (21), in the LV of MLK3-KO mice. We bred MLK3-KO mice to a cardiac myocyte-specific NFAT luciferase (NFAT-Luc) strain which expresses a luciferase transgene under the transcriptional control of a hybrid αMHC promotor-NFATs binding sequence (22). Luciferase activity thus serves as a readout for NFAT activation in the cardiac myocyte (CM). We observed a greater than 2-fold increase of basal Luc activity in both the LV and the isolated CM of the NFAT-Luc^+^ MLK3-KO mice, compared with NFAT-Luc^+^ MLK3 WT littermates (Figure 3), indicating increased cardiac myocyte transcriptional activity of NFAT associated with MLK3 deletion. LV expression of other fetal genes, including *nppa* and *nppb*, did not differ between genotypes (7). The expression of the *Atp2a2* gene, which normally decreases as part of the LV reversion to the fetal gene expression pattern, also was not altered between LV tissue of WT and KO mice (Supplemental Figure 1; supplemental material available online with this article; https://doi.org/10.1172/jci.insight.149075DS1).

### MLK3 contributes to BP control but is not necessary for the hypotensive effects of sildenafil.

**Figure 4:**
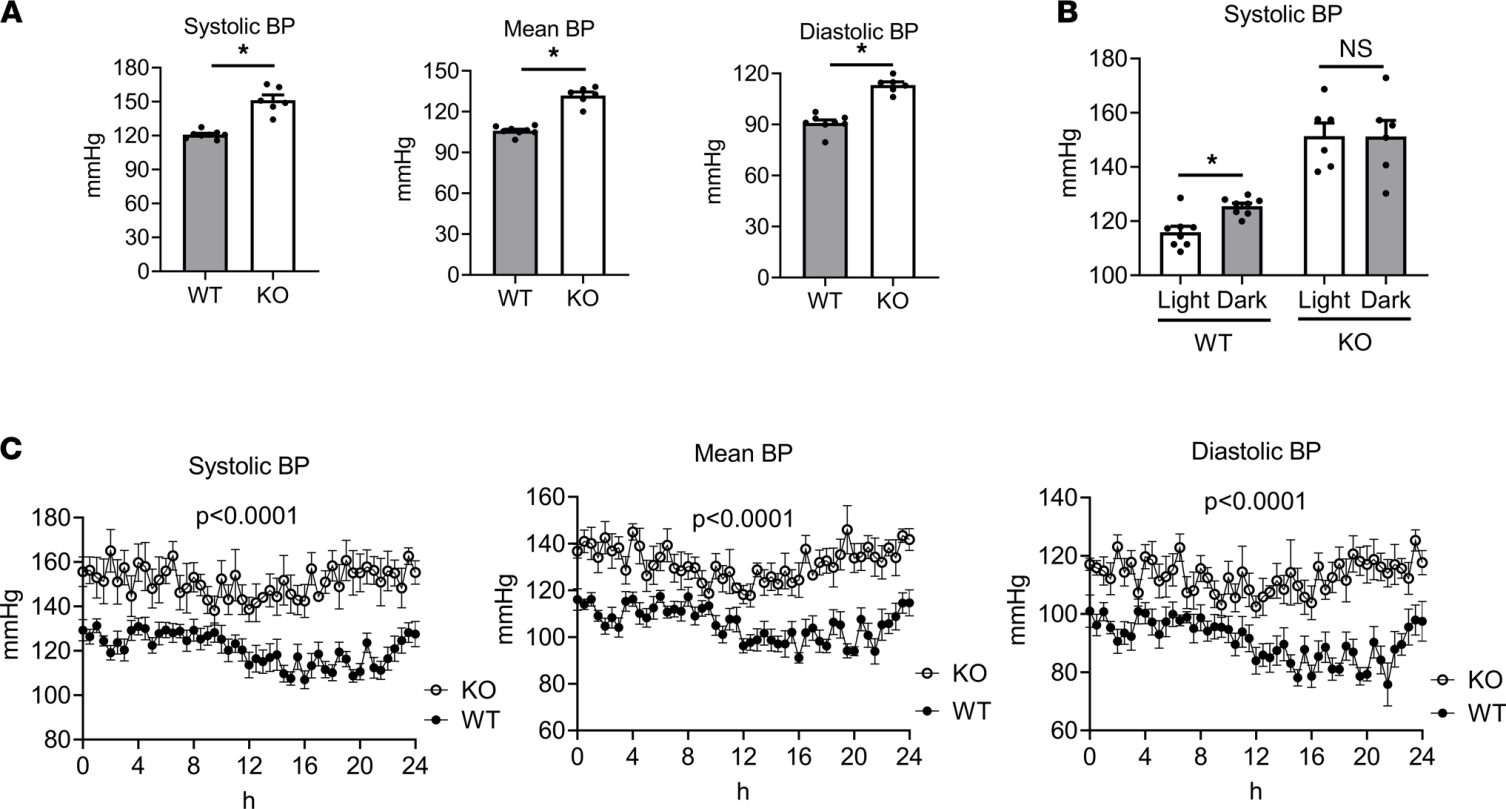
Genetic deletion of MLK3 leads to systemic hypertension in mice. MLK3-KO or WT littermate mice were implanted with arterial telemetric monitors and BP recorded for 24 hours. (**A**) Mean 24-hour systolic, mean, and diastolic BP. *P < 0.0001 by Student’s unpaired 2-tailed *t* test. (**B**) Average light and dark cycle BPs. *P < 0.005 by Student’s paired 2-tailed *t* test. (**C**) Continuous values over 24 hours of systolic, mean, and diastolic BP. The 24-hour data were analyzed by 2-way ANOVA comparing genotype factor. *n* = 8 WT, 6 KO. MLK3, mixed lineage kinase 3.

Because PKG1α LZ–dependent substrates also modulate BP (14), we next examined the role of MLK3 in BP regulation. First, we measured BP by telemetry in adult male MLK3-KO mice compared with WT littermates. Compared with WT, KO mice displayed elevated BP (Figure 4), with increases in systolic, diastolic, and mean arterial pressure. Diurnal variation in BP was also absent in the MLK3-KO mice (Figure 4B). Activity level and heart rate did not differ between genotypes (Supplemental Figure 2). To test whether MLK3 might contribute to BP by a direct impact in the kidney, we first examined MLK3 expression in the kidney compared with other tissues. Whereas we detected MLK3 protein expression in tissue lysates from the LV, aorta, spleen, thymus, and brain, MLK3 protein expression was undetectable in kidney tissue lysates (Supplemental Figure 3). To determine the contribution of excessive sodium retention to the elevated BP in MLK3-KO mice, we tested the BP response of KO mice to a reduction in dietary sodium. One-week exposure to a low-sodium (0.02 %) diet resulted in a nonsignificant trend toward decreased BP in KO (11% decrease) and WT littermates (3% decrease). However, BP remained significantly elevated in KO mice compared with WT (Supplemental Figure 4), supporting a renal system–independent component to MLK3 BP regulation.

**Figure 5:**
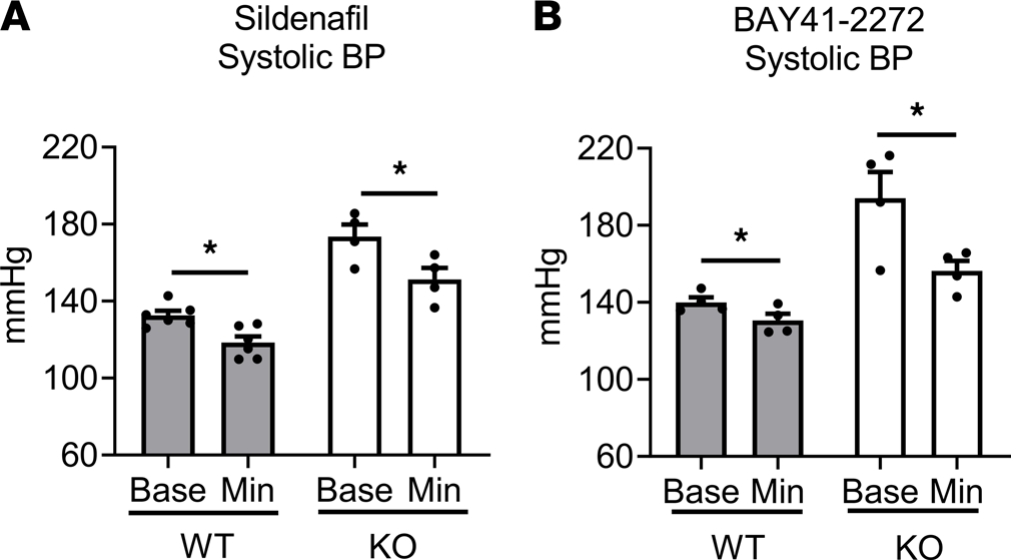
Blood pressure in MLK3 deletion mice is responsive to acute PKG activation with phosphodiesterase 5 inhibition or soluble guanylate cyclase stimulation. Baseline and minimum BPs in male MLK3-KO or WT littermate mice with implantable arterial telemetry after i.p. injection of (**A**) sildenafil (6 mg/kg) or (**B**) the guanylate cyclase stimulator BAY41-2272 (8 mg/kg). *n* = 6 WT, 4 KO. *P < 0.05 by paired 2-tailed *t* test. MLK3, mixed lineage kinase 3.

We next tested whether MLK3, as a PKG1α substrate, mediated the acute hypotensive effects of PKG1 activation. In mice implanted with BP telemeters, we administered the PDE5 inhibitor sildenafil or the soluble guanylate cyclase stimulator BAY41-2272 to test 2 different mechanisms of PKG activation. As expected, both BAY41-2272 and sildenafil administration reduced BP in WT mice (Figure 5). Unexpectedly, both PKG activators significantly decreased BP in KO littermates. Thus, these data indicate that MLK3 was not necessary for the effect of cGMP via PKG1α to lower BP acutely in vivo.

### MLK3 genetic deletion leads to increased vascular stiffness, reduced resistance arteriolar distensibility, and abnormalities of vascular smooth muscle cells.

**Figure 6:**
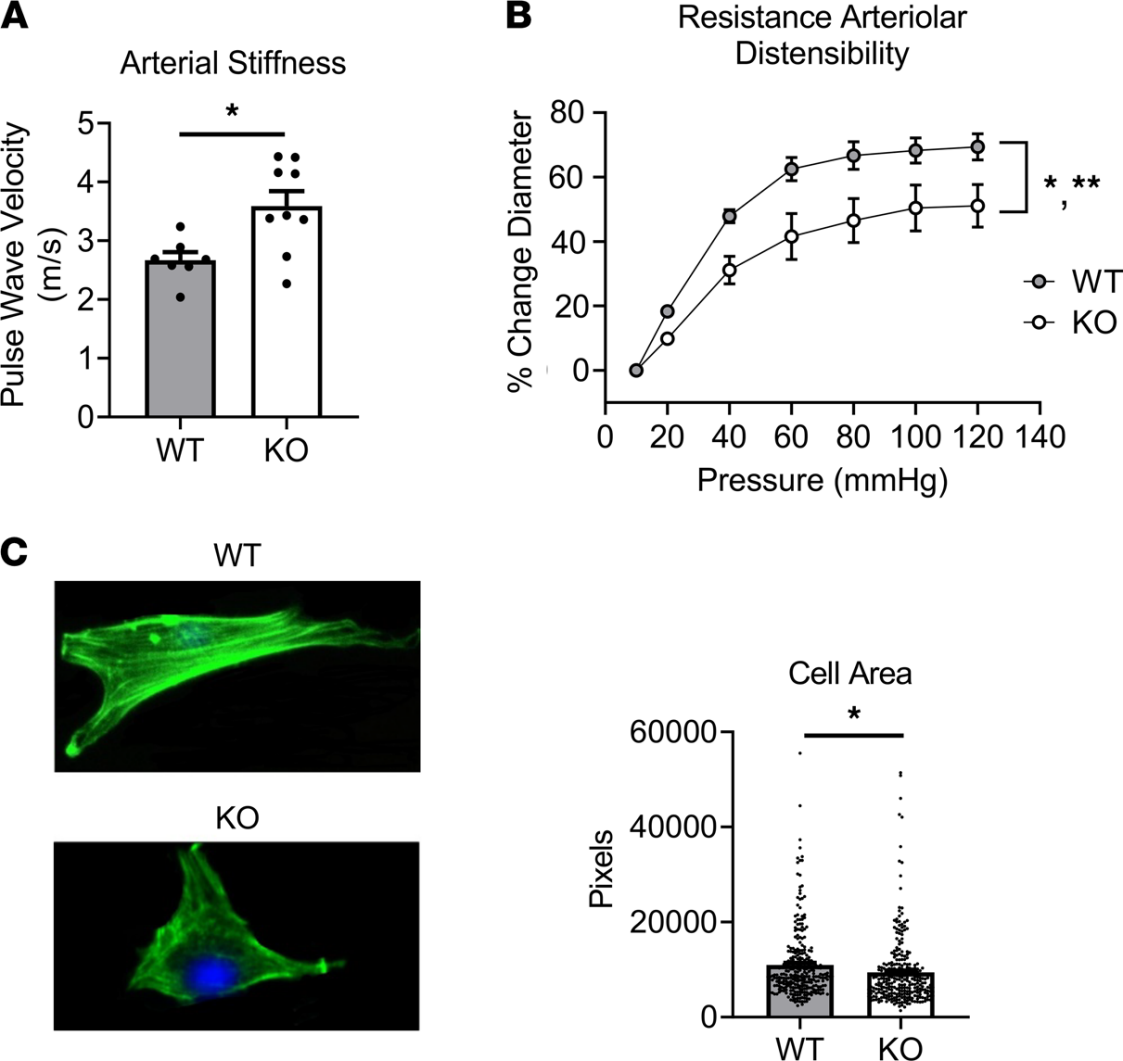
MLK3 genetic deletion leads to increased vascular stiffness, reduced resistance arteriolar distensibility, and primary abnormalities of VSMCs. (**A**) Arterial stiffness determined by aortic pulse wave velocity from MLK3-KO or WT littermate mice. *n* = 7 WT, 9 KO. *P < 0.05 by Student’s unpaired 2-tailed *t* test. (**B**) Distensibility calculated from pressure myography of ex vivo mesenteric resistance arterioles from MLK3-KO or WT mice. *n* = 3 per genotype. *P < 0.05 main effects of genotype; **P < 0.01 for interaction of genotype with pressure factor, by 2-way ANOVA. (**C**) Representative image and quantitation of cell area in freshly dispersed and fixed primary aortic SMCs isolated from MLK3-KO mice and WT littermates stained with phalloidin and DAPI counterstain. Original magnification, ×200, for all images. Representative of 3 independent experiments. *P < 0.05 by Student’s 2-tailed *t* test. *n* = 250 cells per genotype. MLK3, mixed lineage kinase 3; VSMCs, vascular smooth muscle cells.

We next investigated arterial and arteriolar stiffness as a vascular mechanism contributing to hypertension in MLK3-KO mice. MLK3-KO mice have increased vascular stiffness compared with WT controls as measured by aortic pulse wave velocity (Figure 6A). Mesenteric resistance arterioles isolated from MLK3-KO mice also demonstrated reduced distensibility compared with WT littermates, as measured by pressure myography to quantify the change in diameter to passive stretch across a range of pressures (Figure 6B). However, resistance arteriolar wall thickness and collagen fraction did not differ between genotypes (Supplemental Figure 5), suggesting that abnormalities of extracellular matrix or structure did not contribute to the changes in vessel stiffness. By contrast, freshly dispersed aortic smooth muscle cells (SMCs) from KO mice displayed reduced cell area compared with WT controls, as assessed by phalloidin stain (Figure 6C).

### RhoA kinase inhibition normalizes BP in MLK3-deficient mice.

**Figure 7:**
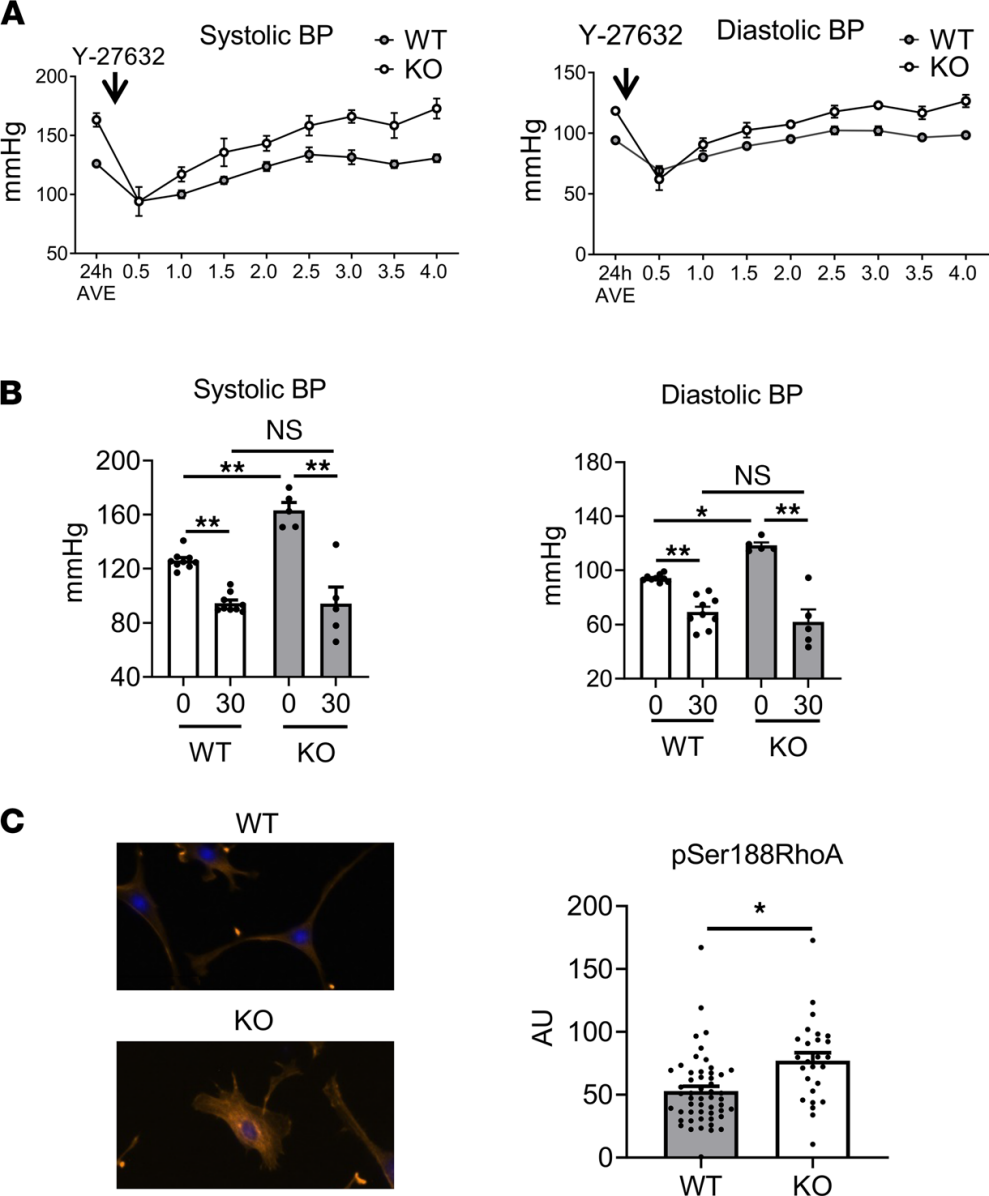
RhoA kinase inhibition normalizes BP in MLK3 deletion mice. (**A**) Systolic and diastolic BPs recorded from male MLK3-KO mice or WT littermates with implantable arterial telemeters after i.p. injection of the RhoA kinase inhibitor Y-27632 (15 mg/kg). (**B**) Systolic and diastolic BPs at baseline and 30 minutes after injection of the RhoA kinase inhibitor Y-27632 in the same mice as in **A**. *P < 0.005, **P < 0.001 by 2-way ANOVA. *P* = 0.0003 for interaction of 30-minute drug treatment with genotype on systolic BP effects; *P* = 0.0013 for interaction of 30-minute drug treatment with genotype on diastolic BP effects. *n* = 9 WT, 5 KO. (**C**) Representative image and quantitation of PKG1α inhibitory phosphorylation of RhoA in freshly dispersed and fixed primary aortic SMCs from WT and MLK3-KO littermates stained with antibody against pSer188 RhoA. Original magnification, ×200, for all images. *P < 0.05 by Student’s 2-tailed *t* test. *n* = 50 WT, 26 KO cells. MLK3, mixed lineage kinase 3; SMCs, smooth muscle cells.

MLK3 inhibits the GTPase RhoA (10) in vascular SMCs (VSMCs; ref. 8). RhoA activation also promotes VSMC stress fiber accumulation, reduction of VSMC area (23), and increased vascular stiffness (24). Because the MLK3-KO vessels and VSMCs displayed these hallmarks of RhoA activation, we tested the contribution of RhoA pathway disinhibition to the BP phenotype of the MLK3-KO mice. Consistent with prior findings that RhoA activity is increased in MLK3-KO VSMCs, the RhoA kinase inhibitor Y-27632 reduced systolic and diastolic BP in the KO mice after 30 minutes, eliminating the BP difference between KO and WT mice (Figure 7, A and B). However, MLK3 deletion did not interfere with inhibitory phosphorylation of RhoA on the PKG-specific (2) Ser188; rather RhoA phosphorylation was increased in KO VSMCs, supporting that MLK3 was not required for basal PKG1α phosphorylation of RhoA in VSMCs (Figure 7C).

### MLK3 kinase inhibition does not acutely affect BP but reduces LV function.

**Figure 8:**
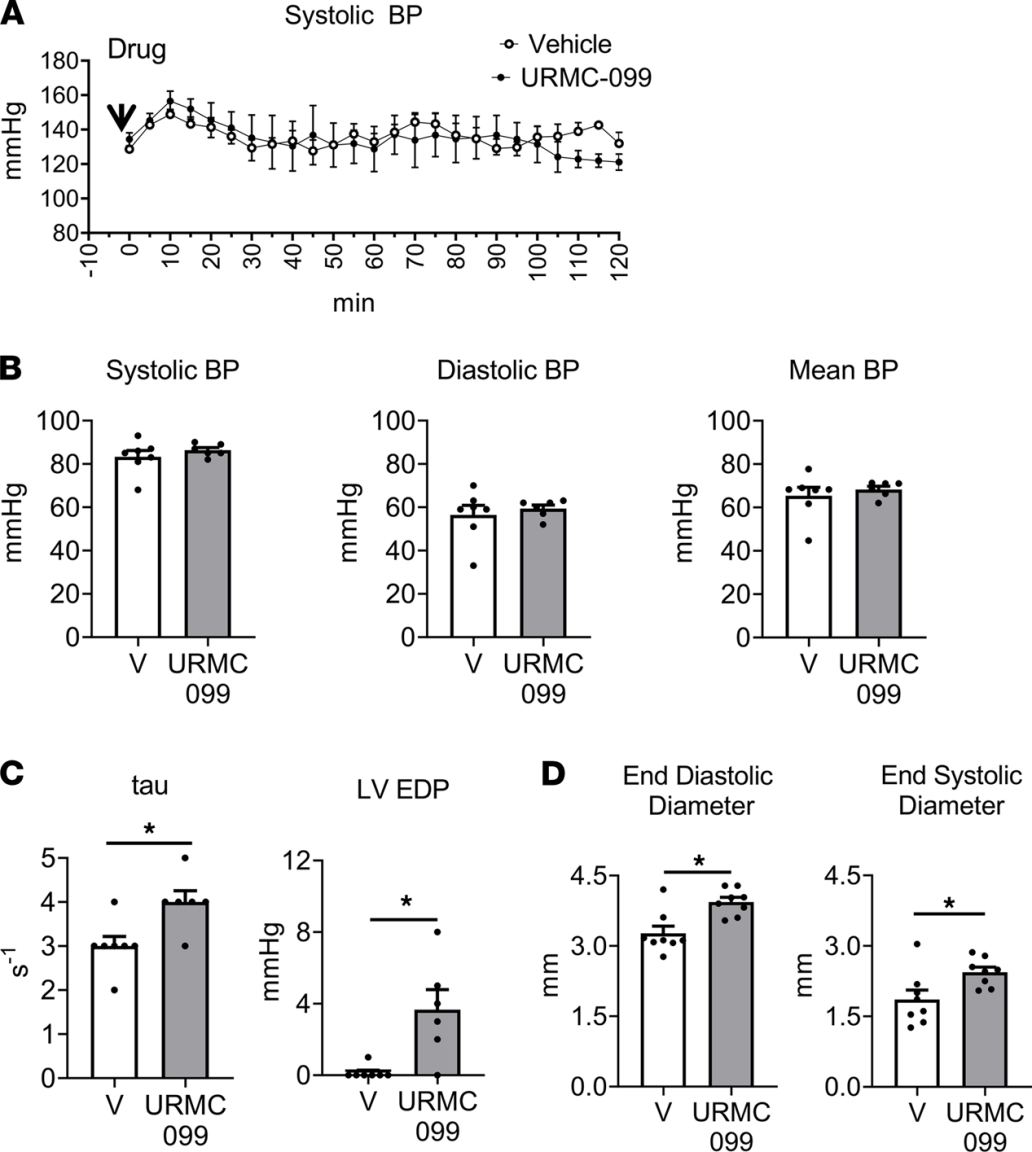
MLK3 kinase inhibition induces left ventricular dilation and diastolic dysfunction in mice in the absence of BP effects. (**A**) Systolic BP recorded from WT mice with implantable arterial telemeters after i.p. injection of the MLK3 inhibitor URMC-099 (10 mg/kg) or V control. *n* = 4 per treatment. (**B** and **C**) Invasive hemodynamics were performed in mice treated for 14 days with URMC-099 10 mg/kg twice daily by i.p. injection to measure (**B**) systolic, diastolic, and mean BPs, and (**C**) LV time constant of LV relaxation (tau), and LV EDP; *n* = 8 vehicle, 6 URMC-099. (**D**) Left ventricular end diastolic and end systolic dimensions measured by echocardiography in male mice treated for 14 days with URMC-099 or V control; *n* = 8 V, 8 URMC-099. *P < 0.05 by Student’s 2-tailed *t* test. MLK3, mixed lineage kinase 3; LV, left ventricle; EDP, end diastolic pressure; V, vehicle.

MLK3 inhibits RhoA through kinase-independent mechanisms (10). To investigate the contribution of MLK3 kinase function to BP regulation, we administered the MLK3 inhibitor URMC-099 in conscious mice implanted with arterial telemeters. Administration of URMC-099 had no acute effects on BP over 120 minutes (Figure 8A). We next tested the effects of URMC-099 on cardiovascular parameters in vivo. We administered URMC-099 for 14 days, a duration that has previously been established to abolish chronic MLK3 signaling in vivo (25). In this sustained model, treatment with URMC-099 (10 mg/kg twice daily; ref. 25) also did not alter BP compared with vehicle treatment (Figure 8B), as measured in invasive hemodynamic studies. However, 14-day MLK3 inhibition induced worsening diastolic function as measured by tau, the time constant of LV relaxation, and increased LV end diastolic pressure (Figure 8C). URMC-099 administration also increased LV chamber sizes compared with vehicle-treated littermates (Figure 8D).

## Discussion

**Figure 9:**
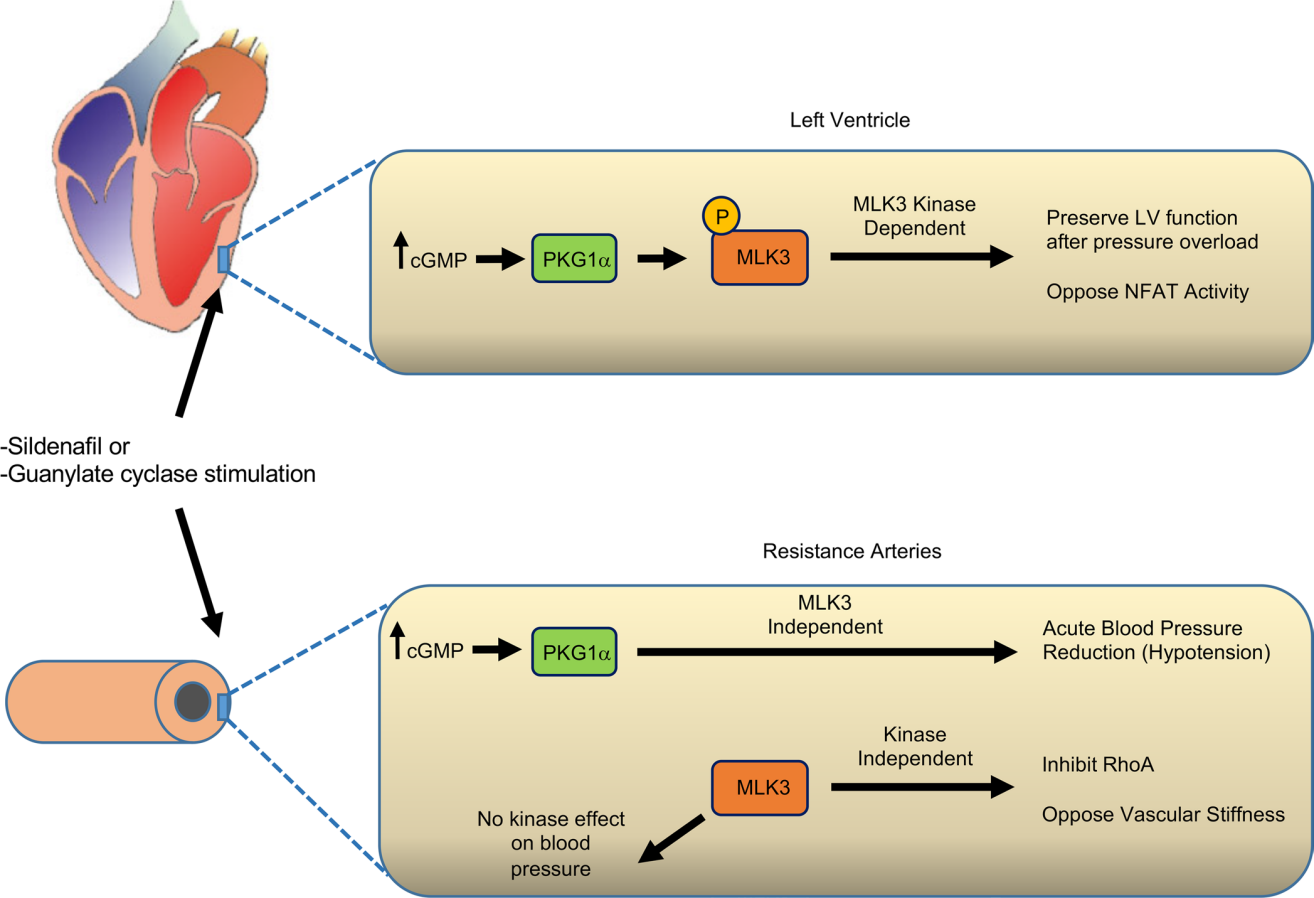
Proposed model. PKG1α binds and phosphorylates MLK3 leading to increased MLK3 kinase activation. In the LV, PKG1α activation of MLK3 promotes LV functional compensation to pressure overload. In the vascular smooth muscle cell, MLK3 inhibition of RhoA opposes vascular stiffening, but is not required for PKG1-induced acute BP reduction. PKG1α, cGMP-dependent protein kinase 1α; MLK3, mixed lineage kinase 3; LV, left ventricle.

In the current study, we investigated the contribution of MLK3 to PKG1 regulation of LV function and BP. We demonstrate that (a) MLK3 functioned as a PKG1α-interacting partner and kinase substrate; (b) MLK3 was required for therapeutic effects of sildenafil on LV function after 7-day pressure overload; (c) MLK3 deletion led to hypertension and increased vascular stiffness in mice; (d) MLK3 was not required for the pressure lowering effects of PKG1α stimulation; and (e) pharmacological inhibition of MLK3 kinase activity induced LV dysfunction but did not increase BP. Taken together these findings identify a model (Figure 9) in which MLK3 mediated PKG1α-induced LV compensation to pressure overload, but MLK3 controlled BP through inhibition of RhoA signaling in the VSMC, which is independent of PKG1α-mediated acute regulation of BP.

Here we demonstrate that PKG1α coimmunoprecipitated with MLK3 in LV tissue via the PKG1α LZ domain and that PKG1α induced phosphorylation of the MLK3 kinase activation domain. We interpret these findings to identify MLK3 as a PKG1α LZ–dependent substrate in the myocardium. We previously identified a critical role of the PKG1α LZ domain in preserving LV function in pressure overload (1). The PKG1α LZ domain is required for PKG1α binding to important effectors in the cardiovascular system (13, 15) and specifically in the CM, including regulator of G protein signaling 2 (RGS2; ref. 26), troponin T (27), and cardiac myosin binding protein-C (16). These findings add MLK3 as another PKG1α LZ domain–binding partner and may help explain why both MLK3 and PKG1α are required for myocardial JNK activation during the compensatory phase of LV pressure overload (1, 7). Indeed, genetic deletion of MLK3 (7) and disruption of the PKG1α LZ protein interaction domain (1) each lead to impaired LV function within 7 days of TAC. This study supports that they act in the same pathway, as sildenafil-induced LV protection was lost in MLK3-KO mice.

To our knowledge, these findings provide the first evidence of MLK3 regulation by a cardioprotective kinase. Thus, other cardiovascular regulators of MLK3 phosphorylation state and function may be of interest. Other endogenous modulators of MLK3 include TNF receptor–activated factor 2 (TRAF2; ref. 28) and the small GTPase cdc42 (29), each of which induce MLK3 activation through allosteric cointeractions with MLK3. TRAF2 and cdc42 each preserve LV function in the setting of exogenous stress (30, 31) and activate MLK3 in noncardiovascular cells. However, direct regulation of MLK3 by these proteins in the cardiovascular system has yet to be investigated. Understanding the precise molecular basis of MLK3 activation could provide peptide or small molecule-based strategies which could be tested as therapeutic approaches for HF.

The reduced cointeraction of PKG1α and MLK3 in the failing LV further supports the importance of this interaction. In pressure overload, PKG1α association with other antiremodeling substrates, such as RGS2, decreases (26). Further, pressure overload–induced direct oxidative modifications of PKG1α alter its intracellular localization in the CM and disrupt normal substrate interactions (32). We interpret our current findings to support a mechanism in which PKG1α normally binds and activates MLK3 in LV tissue and in which disruption of PKG1α-MLK3 cointeraction promotes LV dysfunction in pressure overload. Future studies will be needed to test the degree to which phosphorylation of the MLK3 ser281 site is necessary for the PKG1 effect on the LV response to TAC.

Our finding that sildenafil failed to improve LV function in MLK3-KO mice after TAC demonstrates the requirement of MLK3 for the functional effects of PKG1α in vivo. We chose the 7-day TAC model because prior studies demonstrate critical roles of the PKG1α LZ domain, and of LZ-dependent substrates, in the initial compensatory stages of LV pressure overload. For example, disrupting mutation of the PKG1α LZ domain in mice leads to high mortality and LV dysfunction within the first 7 days after TAC. Genetic deletion of RGS2, a PKG1α LZ–dependent effector (14), also produces accelerated HF, LV dysfunction, and death in mice (26). Moreover, our prior published work identified that a robust reduction of the therapeutic effect of sildenafil in mice with mutation of the PKG1α LZ domain occurs at 7 days after TAC (1). We therefore posited that this time point would be the most relevant to assess effects of MLK3 deletion on sildenafil therapeutic effects in TAC. Finally, the PKG1α LZ domain mediates myocardial JNK activation after TAC (1), and multiple studies support a role of JNK and its upstream activators in the myocardium as primarily promoting LV compensation within the first 14 days of pressure overload (33–36). Our prior work has shown that MLK3 is necessary for myocardial JNK activation within the first week of TAC (7). These combined data informed our rationale for focusing on the role of MLK3 in mediating initial functional effects of sildenafil in pressure overload. Future studies will investigate the potential requirement of MLK3 for the long-term benefits of PKG1 activation on chronic cardiac remodeling.

Our findings of impaired sildenafil effect in MLK3-KO TAC mice are of interest because other PKG1-activating drugs such as nitrates and vericiguat have improved outcomes in HF with reduced ejection fraction, but in some cases their efficacy has been unsustained (37) or relatively modest (5). A large body of mechanistic and human studies supports that disruption of endogenous cGMP activation of PKG1α and altered PKG1α substrate access promotes LV dysfunction and HF (1, 26, 32, 38–40). In the failing LV, these maladaptive processes may also limit the efficacy of PKG1-activating drugs (reviewed in ref. 38). Together, our findings thus identify disrupted PKG1α interaction with MLK3 as a mechanism through which dysregulation of endogenous PKG1 signaling may have promoted LV failure and reduced the efficacy of PKG1-activating drugs. It will also be interesting to test the cointeraction of MLK3 and PKG1α in human LV tissue, and in other experimental models of HF, and to determine the effect of PKG1 activation on this interaction in HF patients.

We do note that although sildenafil augments intracellular cGMP and thus activates PKG1 in the myocardium (17), cGMP modulation of other effectors, such as PDE2 (41), also promotes inotropy through cAMP activation of protein kinase A. Thus, it remains possible that MLK3 also mediates LV compensation to pressure overload through this mechanism. Finally, it remains a formal possibility that the lack of sildenafil effect in the MLK3 deletion mice arises due to general pathology from chronic global MLK3 deletion, rather than from a specific signal from PKG1α through MLK3. However, the normal baseline LV function of MLK3-KO mice (7) argues against this.

Our observation of hypertension in conscious MLK3-KO mice represents a second principal finding of this study. We examined BP in MLK3-KO mice given the established critical role of the PKG1α LZ domain and LZ-dependent substrates in the regulation of BP (2, 13, 15). PKG1α controls BP through LZ-dependent regulation of vascular relaxation (2), and mutation of the LZ domain prevents both NO or natriuretic peptide–mediated vascular relaxation and reduction of BP (2, 42). We therefore predicted that MLK3 deletion would prevent acute BP reduction by cGMP-generating compounds sildenafil and BAY41-2272, but in fact we observed that these agents reduced BP normally in the KO mice. We conclude that MLK3 functions as a regulator of BP. Further, we interpret our findings to indicate that MLK3 did not mediate acute BP responses induced by PKG1 activation but rather regulated BP by a PKG-independent mechanism. The increased PKG1α phosphorylation of RhoA on Ser188 in VSMCs from MLK3-KO mice further supports that MLK3 was not required for PKG1α-dependent inhibitory phosphorylation of RhoA in VSMCs. That MLK3 deletion actually led to increased PKG1 phosphorylation of RhoA in VSMCs suggests a compensatory negative feedback mechanism in which increased RhoA activation arising from MLK3 deletion in VSMCs induced increased PKG1α inhibitory phosphorylation of RhoA.

We identify increased vascular stiffness in MLK3-KO mice and demonstrate that RhoA kinase inhibition improved BP in these mice. Prior work has established that MLK3 inhibits the small GTPase RhoA (10) and that MLK3 deletion leads to tonically increased RhoA GTPase in VSMCs in vivo (8). Disinhibition of RhoA activity induces increased SMC and vascular stiffness (24, 43). Our findings of increased aortic pulse wave velocity and reduced resistance vessel distensibility now identify that the established RhoA hyperactivation in MLK3-KO mouse arterioles (8) is associated with increased vascular stiffness and hypertension. Our observation that RhoA kinase inhibition rapidly normalizes BP in the MLK3-KO mice demonstrates in vivo that disinhibition of RhoA is a major contributor to the hypertension in this model, and thus that MLK3 tonic inhibition of RhoA normally represses increases in BP. In humans, a single nucleotide polymorphism at the *map3k11* MLK3 gene locus (44) correlates with aging-associated increases in BP and arterial stiffness. These clinical observations, combined with our experimental findings, suggest the causal relevance of MLK3 to hypertension in humans. Taken together, we interpret these findings to indicate that MLK3 normally controlled BP and vascular stiffness through tonic inhibition of RhoA pathway signaling in vivo but did not mediate acute BP-lowering effects of PKG1 (Figure 9).

In the current study we show that MLK3 inhibition with URMC-099 promotes LV dysfunction in vivo in the absence of BP effects. Others previously observed that MLK3 inhibition of RhoA occurs through mechanisms independent of MLK3 kinase function, in which MLK3 allosterically inhibits the RhoA guanine exchange factor p63 (10). By contrast, MLK3 inhibition with URMC-099 disrupts JNK activation and induces cellular hypertrophy in isolated CMs, supporting that the kinase function of MLK3 normally opposes CM hypertrophy (7). In cell culture, MLK3 kinase activity represses activation of the hypertrophic transcription factor NFATs (ref. 21). The increased NFAT activity we observed in MLK3-KO LVs and CMs provides evidence that MLK3 normally represses pathological NFAT signaling in the CM. The presence of increased NFAT activity in the absence of other fetal gene reexpression in MLK3-KO LVs supports a specific effect of MLK3 on NFAT in the cardiac myocyte, which is not completely dependent on BP. Others have shown that JNK directly phosphorylates NFAT, which prevents NFAT nuclear import (45). Based on our findings in the MLK3-KO-NFAT Luc reporter mice, we speculate that MLK3 opposes NFAT nuclear localization through promoting JNK-mediated phosphorylation of NFAT in the CM. We interpret these collective findings to identify that PKG1α activation of MLK3 opposes LV dysfunction through kinase-dependent effects, whereas MLK3 controlled BP through kinase independent mechanisms. Moreover, these findings provide further evidence that at least some of the effect of MLK3 on LV function occurs independently of its BP effects in vivo. It may prove informative to test the combined effects of PKG1 activation and MLK3 inhibition both in the baseline state and in the setting of chronic pressure overload.

Our study has several limitations. First, our in vivo experiments involved whole body genetic deletion or systemic drug administration as opposed to tissue-specific interventions. Therefore, we cannot conclude definitively the cell-type specificity of effects of MLK3 in the regulation of LV function versus BP, and whether the chronic hypertension discovered in the MLK3-KO mice may have contributed to their cardiac response to pressure overload. Additionally, although we have observed derangements in myocardial JNK and NFAT signaling as a result of MLK3 deletion, MLK3 regulates multiple signaling cascades in addition to JNK signaling (9), and thus we acknowledge that additional downstream MLK3 signaling mechanisms may have contributed to the kinase-dependent effects of MLK3 observed in the current study. Finally, the current study focused only on male mice. We focused our investigation on male mice because of the published effects of MLK3 deletion in male mice and due to the differential cardiovascular effects of TAC on male versus female mice (46). We are actively investigating the sex-specific effects of MLK3 in both LV hypertrophy and BP regulation.

Despite these limitations, these findings have specific clinical implications. Activation of PKG has emerged as a clinical strategy for the treatment of HF, with cGMP-augmenting medications, such as nitrates (3, 47), neprilysin inhibitors (4), and guanylate cyclase stimulators (5), demonstrating efficacy in HF with reduced ejection fraction. However, hypotension arising from PKG1-induced acute vasodilation has limited the utility of these drugs (4, 6, 48). Identifying distinct signaling mechanisms that mediate aspects of the PKG1 therapeutic effect in the LV but avoid acute vasodilation and hypotension could identify novel HF treatment strategies to circumvent these limitations. Our current results therefore suggest that strategies to augment MLK3 kinase activity might be investigated to promote LV compensation to pathologic stress but avoid excess hypotension.

In summary, we have identified MLK3 as a PKG1α substrate and as required for in vivo therapeutic effects of PKG1 activation with sildenafil after pressure overload. We also identify a role of MLK3 in the control of BP and vascular stiffness, which functions independently of PKG1α. These findings support a mechanism in which MLK3 modulates separate cardiovascular phenotypes through distinct kinase-dependent versus kinase-independent mechanisms.

## Methods

### Cell culture.

COS-1 and HEK293 cells were obtained from the American Type Culture Collection and cultured in DMEM (Invitrogen) supplemented with 10% FBS, penicillin (100 units/mL), and streptomycin (100 μg/mL). Cells were grown at 37°C in a 5% CO_2_ humidified incubator. Primary aortic SMCs were obtained as described (2) from 10- to 12-week-old mice.

### cDNA expression plasmids.

Full-length WT and LZM *prkg1a* cDNA in a pCI plasmid were transiently transfected in COS-1 cells seeded in 6-well plates for 18 hours with Polyfect Transfection Reagent (QIAGEN) using 1 μg plasmid cDNA per well. pGEX plasmids containing the GST-tagged N-terminal amino acids 1–59 of PKG1α (WT, PKG1α1-59), amino acids 1–236 (WT, PKG1α1-236), or the PKG1α LZM (PKG1αLZM) were used as previously described (15, 16). cDNA encoding FLAG-tagged human MLK3 was in a pCI plasmid, provided by Ajay Rana (Division of Surgical Oncology, University of Illinois at Chicago, Chicago, Illinois, USA).

*Endogenous PKG1α-MLK3 protein–protein interaction studies*. LV protein lysate (1 mg) was resuspended in 1 mL of 1X Cell Lysis Buffer (Cell Signaling Technology) and precleared with Protein G beads (GE Healthcare, 50 μL, 60 minutes, at 4°C). The precleared supernatant was incubated with 4 μg anti-MLK3 antibody (Santa Cruz Biotechnology, MLK3 D-11, sc-166639) and rotated overnight at 4°C. Next, 50 μL Protein G beads were added to the IP mixture (60 minutes at 4°C), and the protein bead complexes were washed 4 times in 1X Cell Lysis Buffer. The resulting protein bead mixture was resuspended in 2X Laemmli sample buffer (MilliporeSigma) and stored at –80°C until assayed by Western blot.

*GST-PKG1αpulldowns in myocardial tissue*. GST-PKG1α pulldowns were performed as described (15, 33). Hearts of C57BL/6 male mice were rapidly excised and snap-frozen in liquid nitrogen, followed by dounce homogenization of the LV in lysis buffer as previously described (1, 15). LV lysates were rocked gently overnight at 4°C with fusion protein beads, followed by washing 3 times in lysis buffer. Proteins were eluted off the beads by boiling for 5 minutes in Laemmli sample buffer, followed by separation by SDS-PAGE and Western blotting for MLK3. Equal loading of fusion proteins was confirmed by Coomassie stain.

*Affinity purification of PKG1αand coincubation with recombinant MLK3*. WT and LZM PKG1α were transiently transfected into COS-1 cells and were affinity purified from 250 μg whole cell protein lysates using cGMP immobilized agarose beads (Biolog) and rotated overnight at 4°C. The cGMP-agarose beads were washed 3 times in tissue lysis buffer (TLB; 20 mM HEPES, 50 mM β-glycerolphosphate, 2 mM EGTA, 1 mM DTT, 10 mM NaF, 1 mM NaVO_4_, 1% Triton X-100, and 10% glycerol). The cGMP agarose beads were resuspended in TLB and incubated with 192 ng recombinant MLK3 (Invitrogen, PV3788) and rotated overnight at 4°C. The protein bead complexes were washed 4 times in TLB. The resulting protein bead mixture was resuspended in 2X Laemmli sample buffer and stored at –80°C until assayed by Western blot.

### Western blotting.

Cells were treated as described in the figure legends, rinsed once in cold PBS, and lysed with TLB supplemented with 1 mM PMSF. LV tissues were pulverized on dry ice and lysed in TLB supplemented with 1 mM PMSF and protease inhibitors (MilliporeSigma, 539134). Lysates were cleared by centrifugation, protein content was quantified by BCA assay, and lysates were diluted in 2X Laemmli sample buffer containing SDS (MilliporeSigma, S-3401). We used the following antibodies in this study: MLK3 (Cell Signaling Technology, 2817, and Abcam, ab51068), P-MLK3 Thr277/Ser281 (Abcam, ab191530), PKG1α (2), and DYKDDDDK (FLAG) Tag (Cell Signaling Technology, 14793). Membranes were incubated with primary antibodies as per the manufacturer’s recommendations and incubated with HRP-linked secondary anti-mouse or anti-rabbit antibodies (GE Healthcare, NA931 and NA934). Membranes were visualized using the ProteinSimple FluorChem E system and images were quantified using Alpha Innotech Imager software.

*PKG1αtarget phosphorylation site prediction*. The human MLK3 protein (MAPKKK11, NCBI reference sequence: NP_002410.1) was entered into the NetPhosK 1.0 server (Center for Biological Sequence Analysis, Technical University of Denmark) to predict protein phosphorylation sites using an unfiltered analysis with a minimum score threshold of 0.50 (19).

### Kinase reactions.

Purified PKG1α protein (Promega, V517A) and recombinant MLK3 protein (Invitrogen, PV3788) were diluted in 20 mM HEPES, 1 mM DTT, and 0.33% Brij-35 and then incubated in a kinase assay buffer (20 mM HEPES, 1 mM DTT, 5 mM MgCl_2_, and 0.1 mM ATP). Proteins were incubated at 30°C for 30 minutes and the reaction was quenched by addition of 2X Laemmli sample buffer.

### VSMC morphology.

Primary aortic SMCs isolated from MLK3 WT and KO littermates were dispersed on gelatin-coated, glass coverslips, followed by fixing with 3.7% paraformaldehyde and permeabilization with PBS and 0.1% Triton X-100. Blocking was performed with 1% BSA in PBS/0.1% Triton X-100. Cells were then labeled with phalloidin conjugated with Alexa Fluor 488 (Invitrogen A12379) and imaged with a Nikon Eclipse fluorescence microscope. Cell area was determined through manual tracing using the NIH ImageJ program.

### Immunofluorescence.

Primary aortic SMCs were fixed and blocked as above and then incubated with phalloidin, as well as with monoclonal antibody against phosphoserine 188 RhoA (Abcam 187027). Cells were labeled next with Cy3-conjugated Affinipure anti-rabbit secondary antibody (Jackson ImmunoResearch, 711-165-152). Cell fluorescence was determined using ImageJ.

### Experimental animals.

Whole body MLK3-KO mice (49) and MLK3 WT littermates on the C57BL/6 background were obtained from breeding MLK3 heterozygote mice. Mice were studied at 3 to 5 months of age unless otherwise noted. Transgenic mice on the FVB background expressing the α-myosin heavy chain–driven NFAT-Luc construct (Myh6/NFAT-luc) were obtained from The Jackson Laboratory (strain 010588). The mice were backcrossed 10 generations on a C57Bl6 background and subsequently crossbred to the MLK3 strain to obtain MLK3 WT; NFAT-luc+ and MLK3-KO; NFAT-luc+ genotypes. Only male mice were used for these studies. Investigators were blinded to animal genotype prior to the animal surgeries and through the subsequent data analysis.

### Ex vivo pressure myography.

Mesenteric resistance arterioles from mice were cannulated in a pressure myograph (Living System Instrumentation) and incubated in calcium-free physiologic salt solution containing 2 mM EGTA and 1 μM sodium nitroprusside for analysis of passive structure over a range of intralumenal pressures (0–180 mmHg) as previously described (50). Distensibility was calculated as the percentage change in lumen diameter (LD) at a given intraluminal pressure (LDp) from LD at 3 mmHg (LDo) as described (50): ([LDp − LDo]/LDo) × 100. After active tone measurements, vessels were superfused with buffer lacking added Ca^2+^ and containing 2 mM EGTA. Passive diameter responses were then recorded over the pressure range 10 to 120 mmHg.

### Quantitative RT-PCR analysis.

Using TRIzol (Invitrogen), mouse total mRNA was extracted, and 1 μg of RNA was reverse-transcribed to cDNA using the QuantiTect Reverse Transcriptase Kit (QIAGEN). Target primers and cDNA samples were incubated in a 384-well plate in triplicate and amplified by quantitative real-time PCR (qRT-PCR) using SsoFast EvaGreen Supermix (Bio-Rad). mRNA expression in LV tissue of WT and KO mice was measured by quantitative PCR (qPCR) as described (7). Primers for qRT-PCR analysis were as follows: *Atp2a2* Fwd CTGTGGAGACCCTTGGTTGT, Rev CAGAGCACAGATGGTGGCTA; *Myh6* Fwd GCCCAGTACCTCCGAAAGTC, Rev GCCTTAACATACTCCTTGTC; *Myh7* Fwd TTTCTGGCGACAAAGACAGGG, Rev AGGGTTAGCCTCGATTTGAT; *Gapdh* Fwd AGGTCGGTGTGAACGGATTTG, Rev TGTAGACCATGTAGTTGAGGTCA. All samples were amplified for 40 cycles performed at 95°C for 15 seconds and 60°C for 1 minute using an ABI Prism 7900 sequence detection system (Applied Biosystems). qPCR data were analyzed using the ΔΔCt method with *Gapdh* as the reference control, and values were normalized to fold change.

### Transthoracic echocardiography.

Mice were initially anesthetized with 2.5% gaseous isoflurane and M-mode images were acquired from the midpapillary short-axis view under 1.0% gaseous isoflurane as previously described (1).

### TAC.

Body weight– and age-matched 10- to 12-week-old littermate mice were randomly assigned to either sham or TAC surgery. TAC was performed as previously described (1, 51) with a 27-gauge needle used to size the ligature around the transverse aorta.

### LV in vivo hemodynamic measurements.

After 7 days of TAC or sham surgery, mice were anesthetized with 2.5% gaseous isoflurane, and hemodynamic analyses were performed using a pressure–volume transducing catheter as described (1). Hemodynamic data were recorded and analyzed using IOX Software (EMKA version 2.1.10).

### Conscious BP recording.

Mice were implanted with arterial telemeters (Data Sciences International, TA11PA-C10). Blood pressure was recorded for 60 seconds every 30 minutes as previously described (2). Mice were maintained on a 12-hour light/12-hour dark cycle, with normal chow (0.3% NaCl; Harlan diet TD8604) and water was available ad libitum. For acute drug administration studies, BP was recorded for 10 seconds at intervals shown in Figure 7 and Figure 8.

### Aortic pulse wave velocity.

Mice were anesthetized with 2.5% isoflurane for ECG recording on a heated platform (37°C) and maintained with approximately 2.0% isoflurane during the procedure to maintain heart rate of 400 to 450 bpm. For pulse wave velocity, the transit time between the proximal and distal abdominal aorta was determined by averaging distances between the foot of the flow waveform and the R-wave of the ECG over 5 cardiac cycles at each location. Pulse wave velocity (mm/ms) was calculated by dividing the distance (mm) by the difference in transit times (ms) obtained at each location as previously described (52).

### Drug administration.

Sildenafil citrate (Pfizer) or vehicle (citrate) was dissolved in drinking water (400 mg/L). For the TAC study, sildenafil administration began the day prior to surgery (day –1). For acute BP studies, sildenafil citrate (MilliporeSigma) was administered by intraperitoneal injection (6 mg/kg). BAY41-2272 (MilliporeSigma) was dissolved in water and administered by i.p. injection as described (53) at 8 mg/kg. The RhoA kinase inhibitor Y-27632 (LC Laboratories) was administered 15 mg/kg by i.p. injection as previously described (54). URMC-099 (Selleckchem) was administered 10 mg/kg by i.p. injection for acute measure of BP and 10 mg/kg twice daily by i.p. injection for 14 days for chronic studies (25).

### Statistics.

Statistics were performed with a 2-tailed unpaired Student’s *t* test, 1 -way ANOVA, or 2-way ANOVA for multiple comparisons, where appropriate and indicated in the figure legends. All data shown represent the results obtained from independent experiments. When multiple replicates of the same biological sample were conducted, this is noted in the figure legends. Data are represented as mean ± SEM. *P* values of less than 0.05 were considered statistically significant.

### Study approval.

All mouse care and procedures were in accordance with and approved by the Institutional Animal Care and Use Committee of Tufts University School of Medicine and Tufts Medical Center.

## Author contributions

TDC, RHK, and RMB designed the research study. TDC, SP, RAUB, SKK, JCM, GLM, KT, ALM, GRW, MJA, WL, JIA, PB, PL, and RMB conducted experiments. TDC, SP, RAUB, SKK, JCM, KT, WL, JIA, PB, PL, DAR, and RMB analyzed data. TDC, RHK, IZJ, and RMB interpreted results. RJD provided key reagents for the study. RMB drafted the manuscript. All authors made edits to the manuscript. Authorship order of the 3 equal contributors was assigned by number of figures to which the author contributed experimentally.

## Supplementary Material

Supplemental data

## Version 1 07/29/2021

In-Press Preview

## Version 2 09/22/2021

Electronic publication
