# Supplementary material for: MLK3 mediates impact of PKG1α on cardiac function and controls blood pressure through separate mechanisms
Source: JCI Insight. 2021 Sep 22;6(18):e149075. doi: 10.1172/jci.insight.149075 (PMC8492323; doi:10.1172/jci.insight.149075)

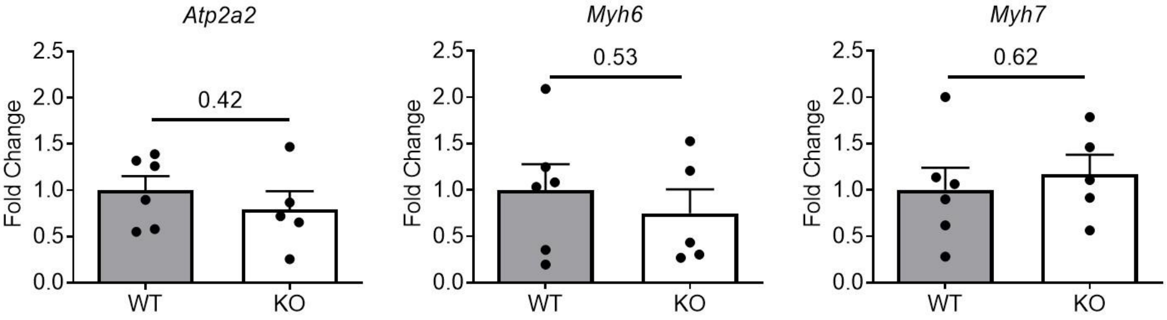

Supplemental Figure 1. Fetal mRNA gene expression in baseline left ventricles of MLK3 KO mice. Expression of mRNA for the *Atp2a2* gene, encoding the SERCA channel, *Myh6* (αMHC, adult isoform), and *Myh7* (βMHC, fetal isoform) normalized to *Gapdh,* in LV tissue from male 12 week-old MLK3 KO mice (n=5) or WT (n=6) littermates, as measured by qPCR. Groups compared by Student's T test. P values displayed.

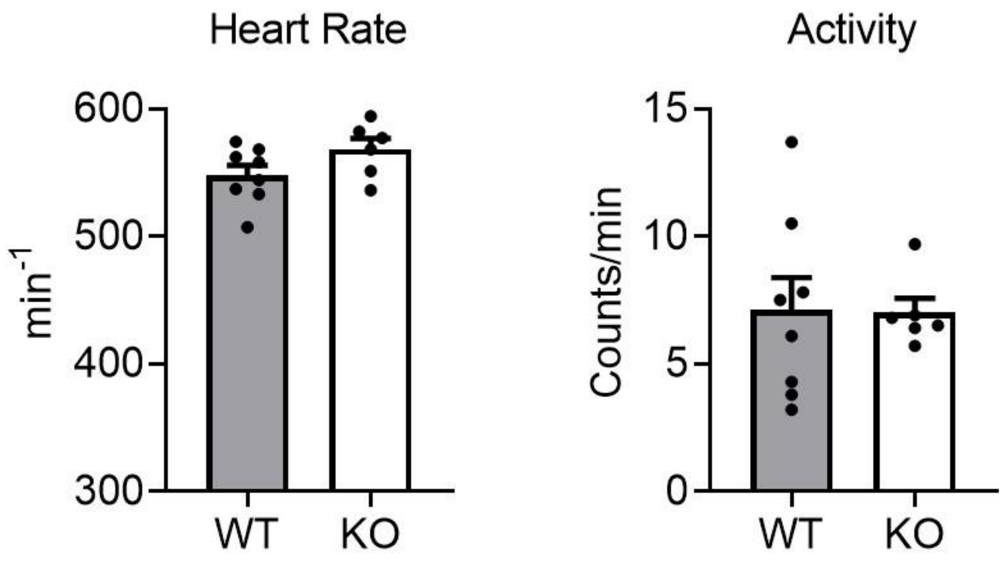

Supplemental Figure 2. Heart rate and activity level in MLK3 KO mice. Telemetric recordings from 12-week old MLK3 KO male mice or WT littermates implanted with arterial telemetric devices. Shown are 24-hour averages of heart rate and displacement. n=8 WT, 6 KO. Data compared by Student's unpaired T test.

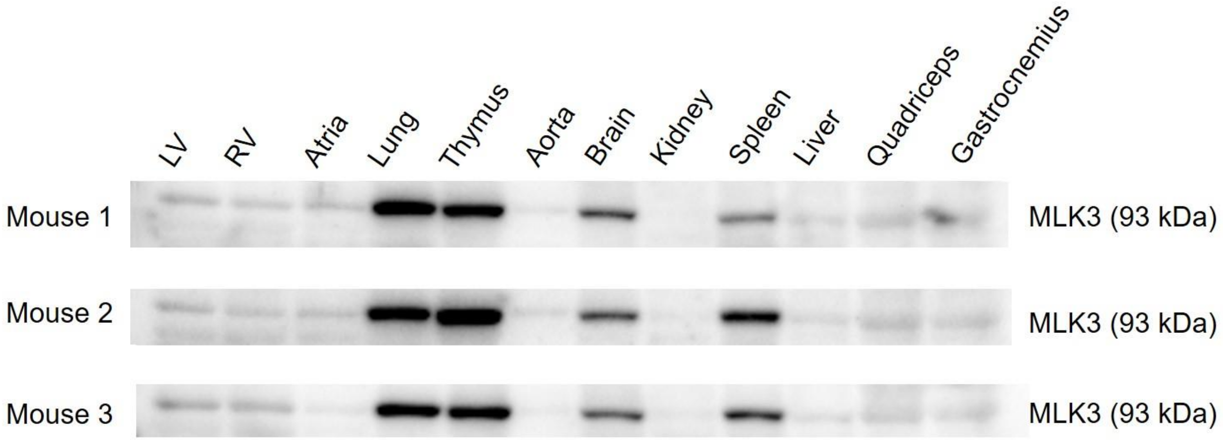

Supplemental Figure 3. MLK3 protein expression in various tissues. Western blot for MLK3 in tissue lysates generated from 3 mice. LV, left ventricle; RV, right ventricle. 50 μg of protein added per lane.

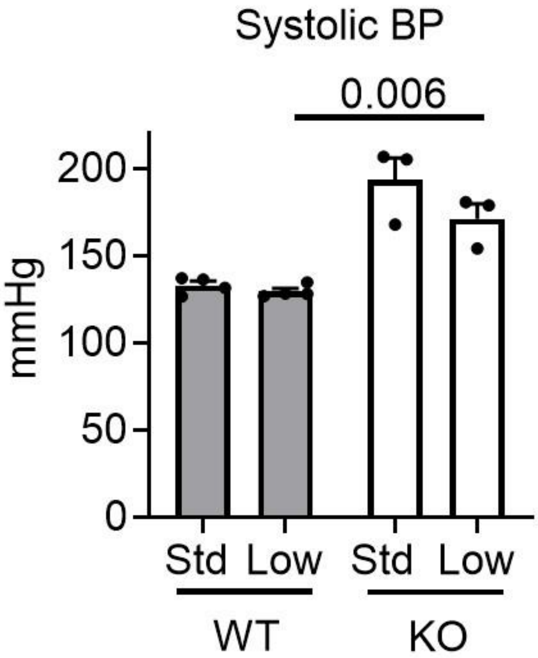

Supplemental Figure 4. Systolic blood pressure of MLK3 KO mice in response to changes in dietary sodium. Average of 24-hour measurement of systolic blood pressure in 4 month old male MLK3 KO or WT littermate control mice implanted with arterial telemeters, on standard sodium (0.3%) diet or after 7 days of low sodium (0.02%) chow. Data analyzed by 1 way ANOVA with Tukey's post-test. n=4 WT, 3 KO mice.

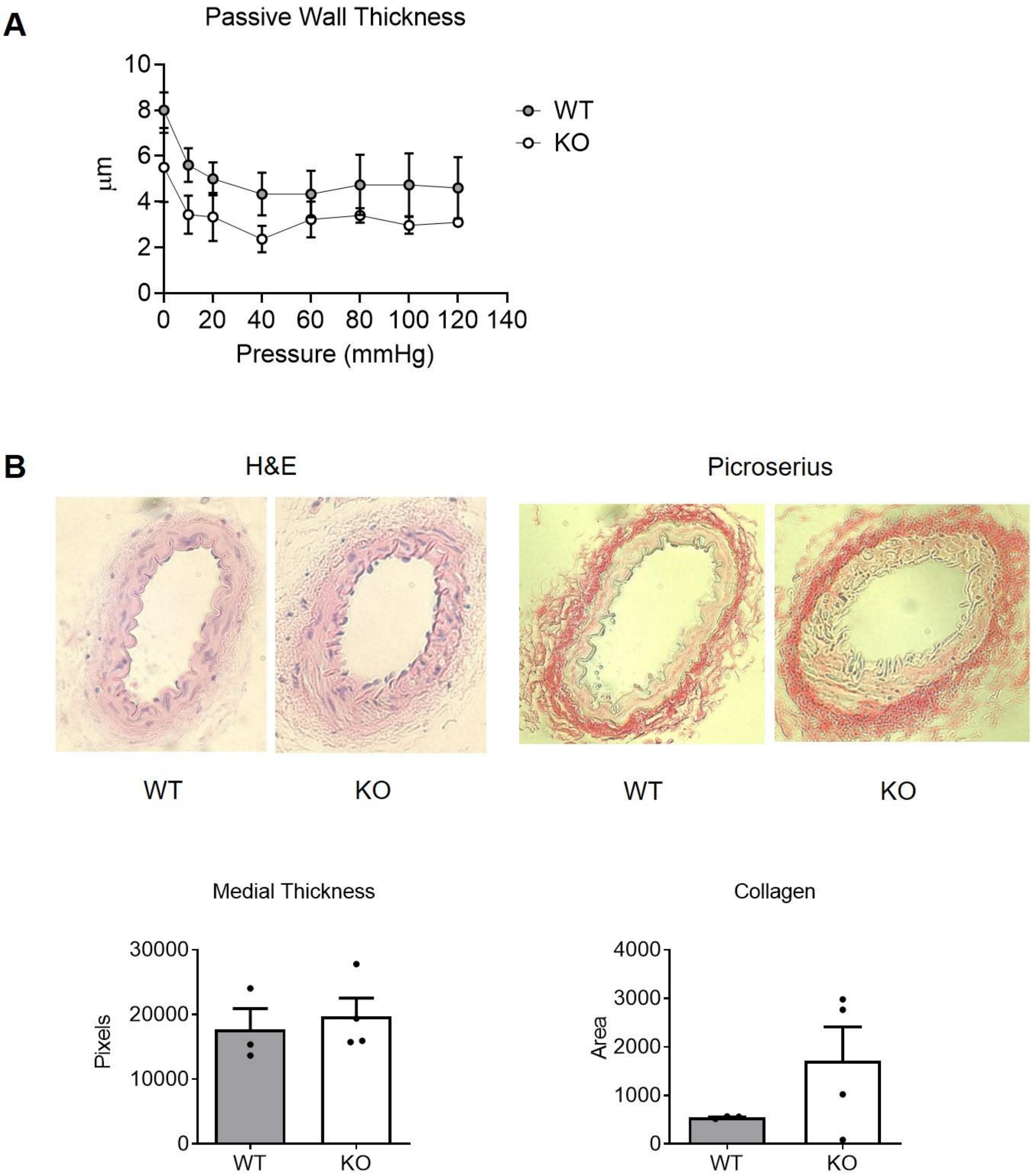

Supplemental Figure 5. Wall thickness and medial collagen content in MLK3 KO arteriolar resistance vessels. (**A**) Wall thickness directly measured by pressure myography in mesenteric resistance arteries isolated from 5 month-old male MLK3 KO or WT littermates, subjected to range of filling pressures. n=3 per genotype. (**B**) representative and summary data of medial thickness by H&E stain and of collagen content by picrosirius red staining in mesenteric resistance arterioles vessels isolated from MLK3 KO male mice or WT littermates. n=3 WT, 4 KO. Data compared by Student’s unpaired T test.

Cropped final Figure 2A

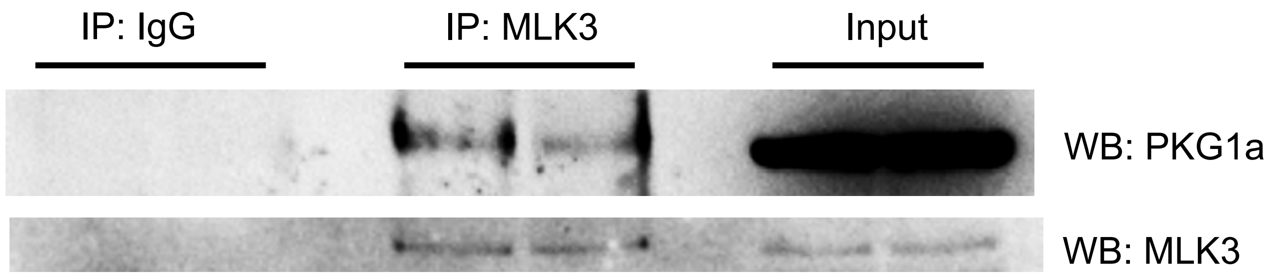

Full unedited gel for Figure 2A: WB for PKGIa

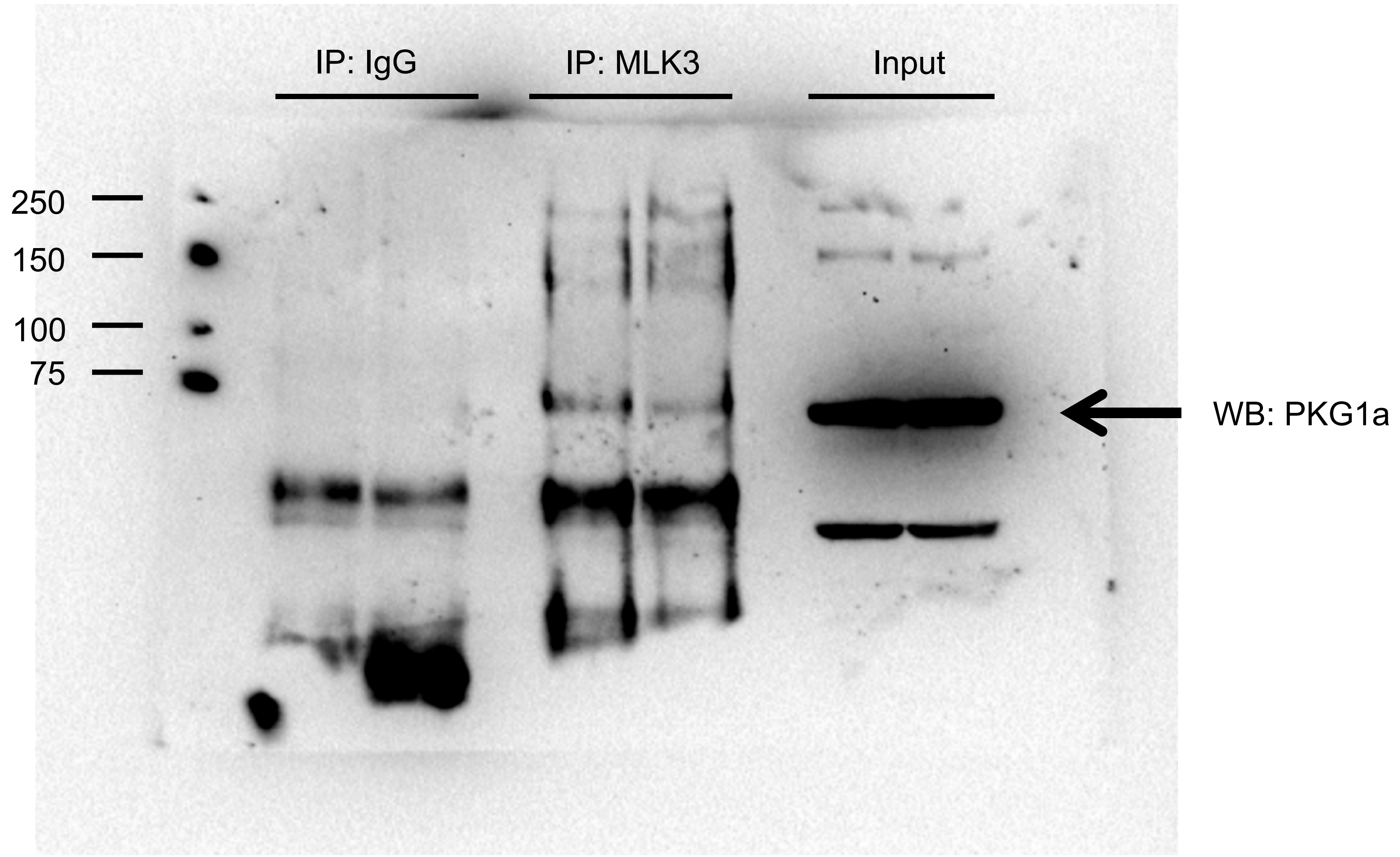

Full unedited gel for Figure 2A: WB for MLK3

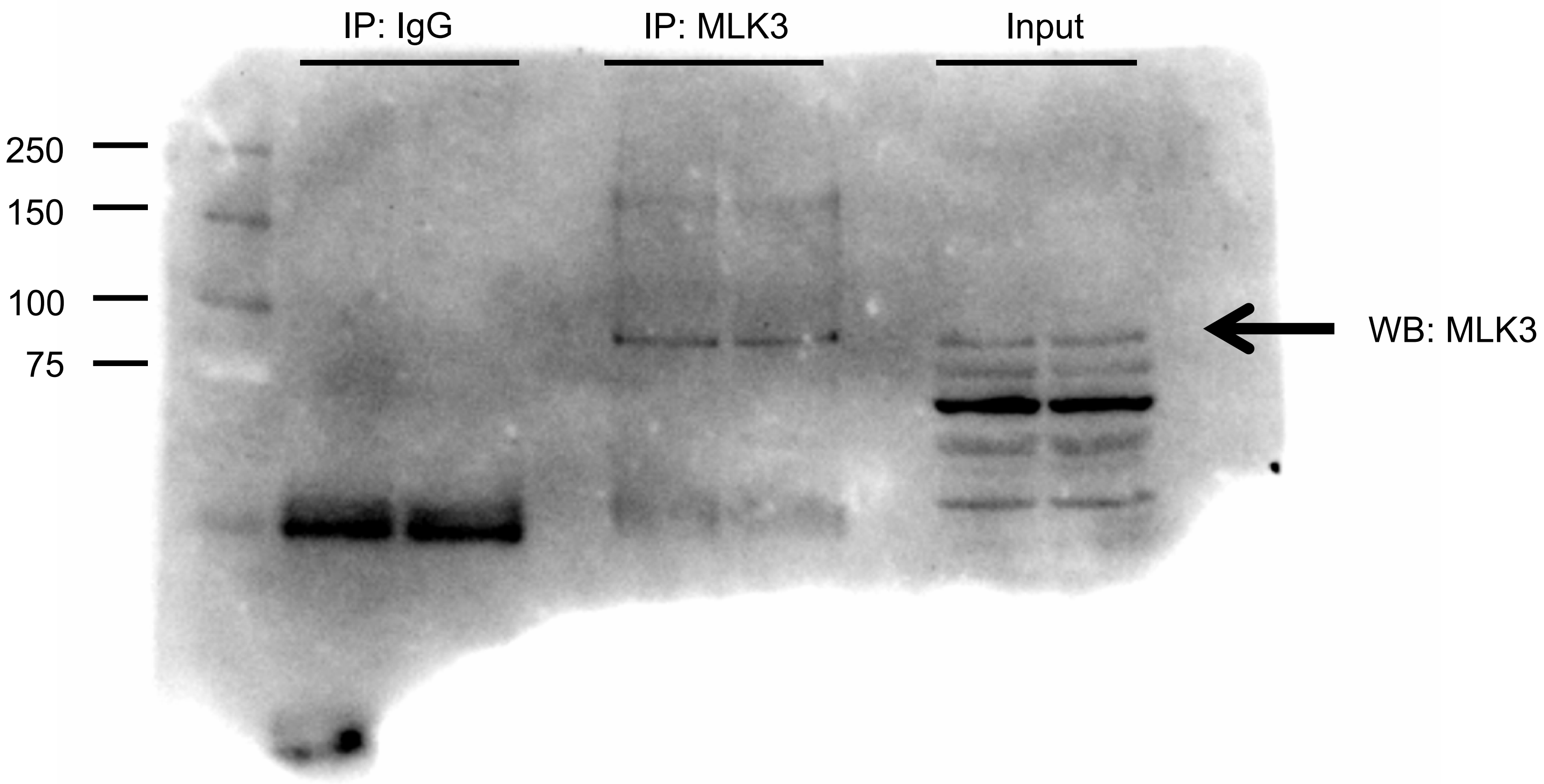

Cropped final Figure 2B

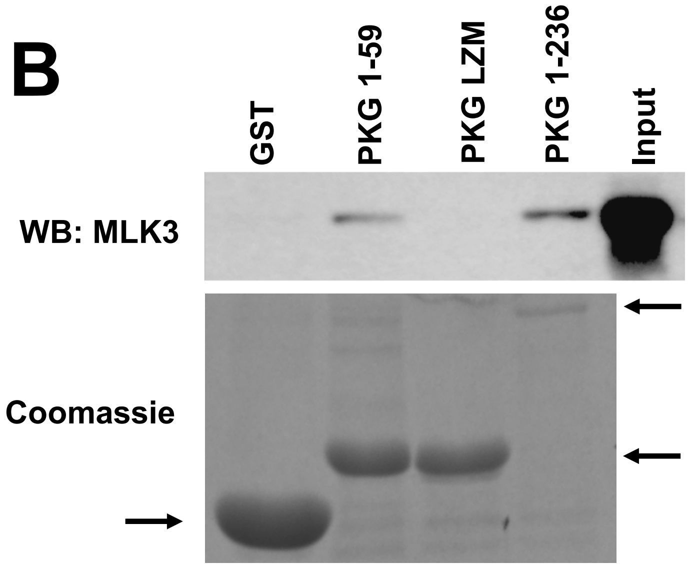

Full unedited gel for Figure 2B: Immunoblot MLK3

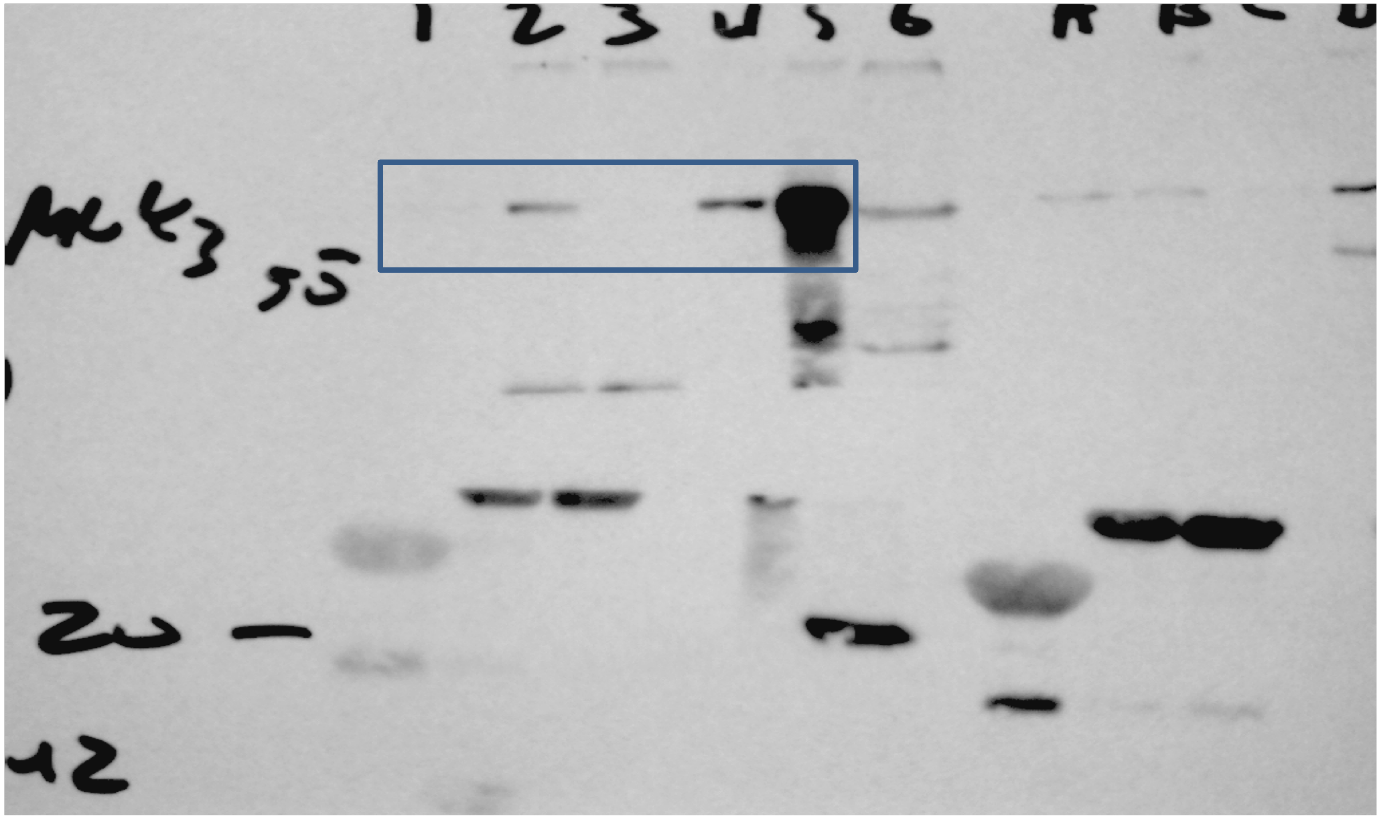

Full unedited gel for Figure 2B: Coomassie Stain of GST Proteins

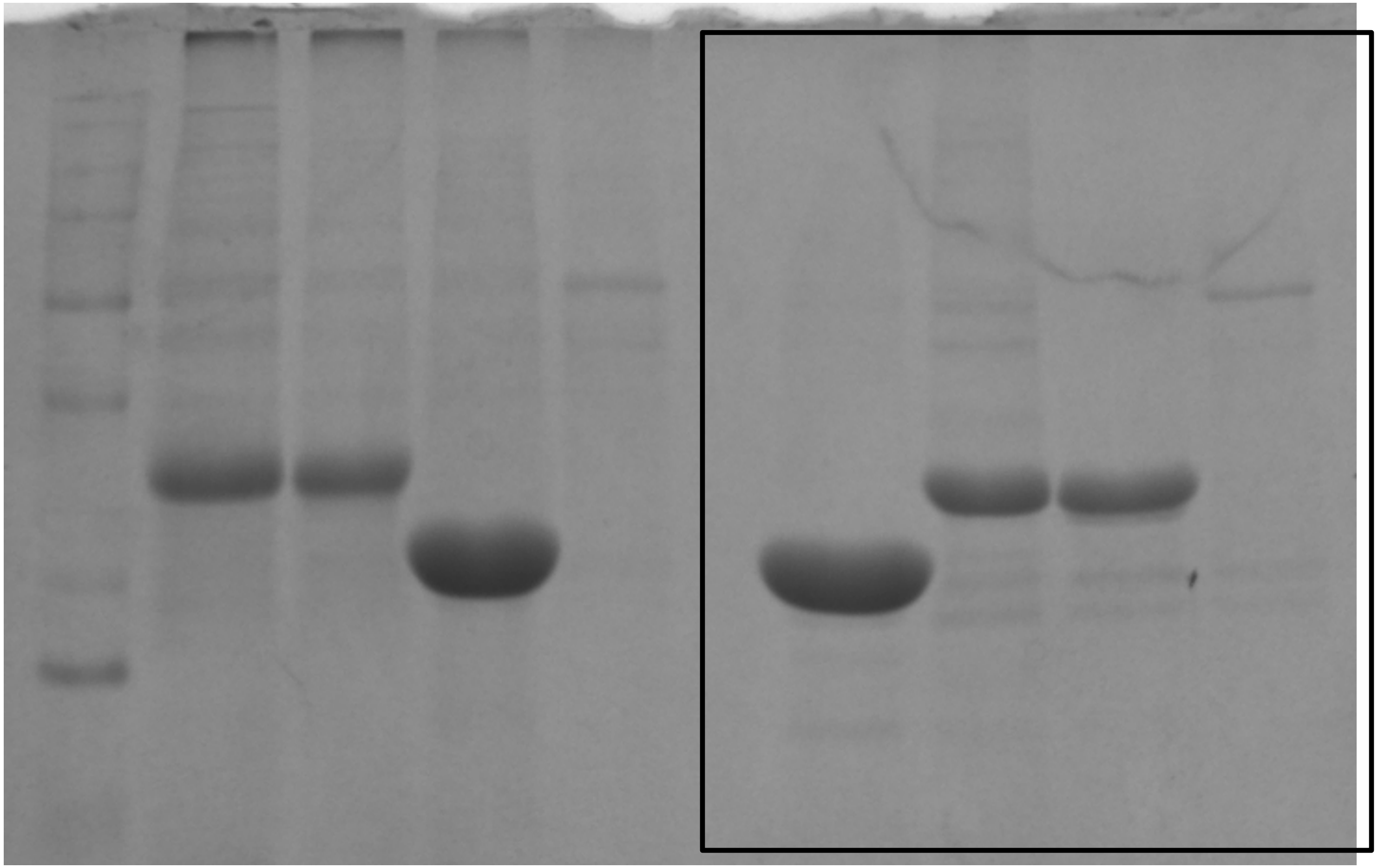

Cropped final Figure 2C

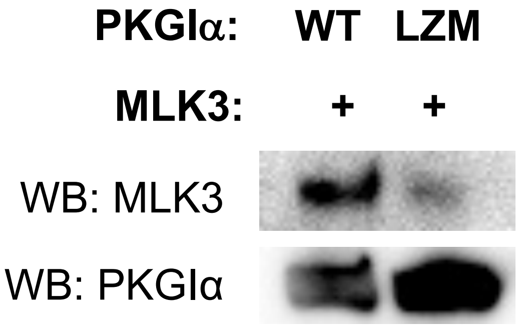

In this experiment PKG1a (WT or LZM) in pCI plasmids were expressed in COS1 cells and affinity purified using cGMP-bound agarose beads. Recombinant MLK3 was added to the protein-bead complex and allowed to incubate before washing and western blotting for co-precipitation.

Cells expressing the different plasmids (pCI, PKG WT, PKG LZM) were compared with or without the addition of recombinant MLK3. A positive control (MLK3 recombinant protein, not co-precipitated) was run the same membrane.

Lanes 5 and 6 were cropped and used in the final image of Figure 1C.

Full unedited gel for Figure 2C: Immunoblot MLK3

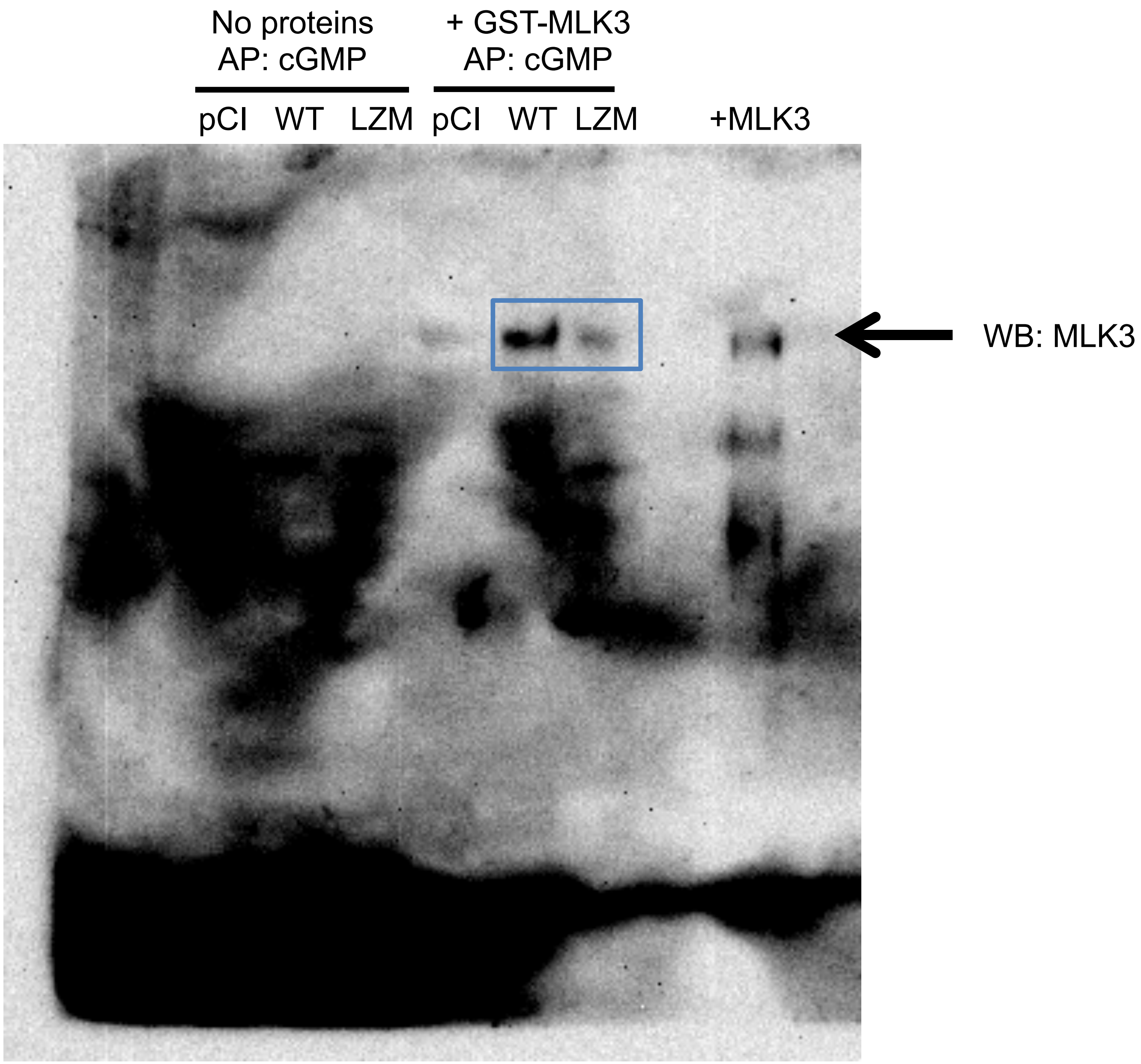

Full unedited gel for Figure 2C: Immunoblot PKGIa

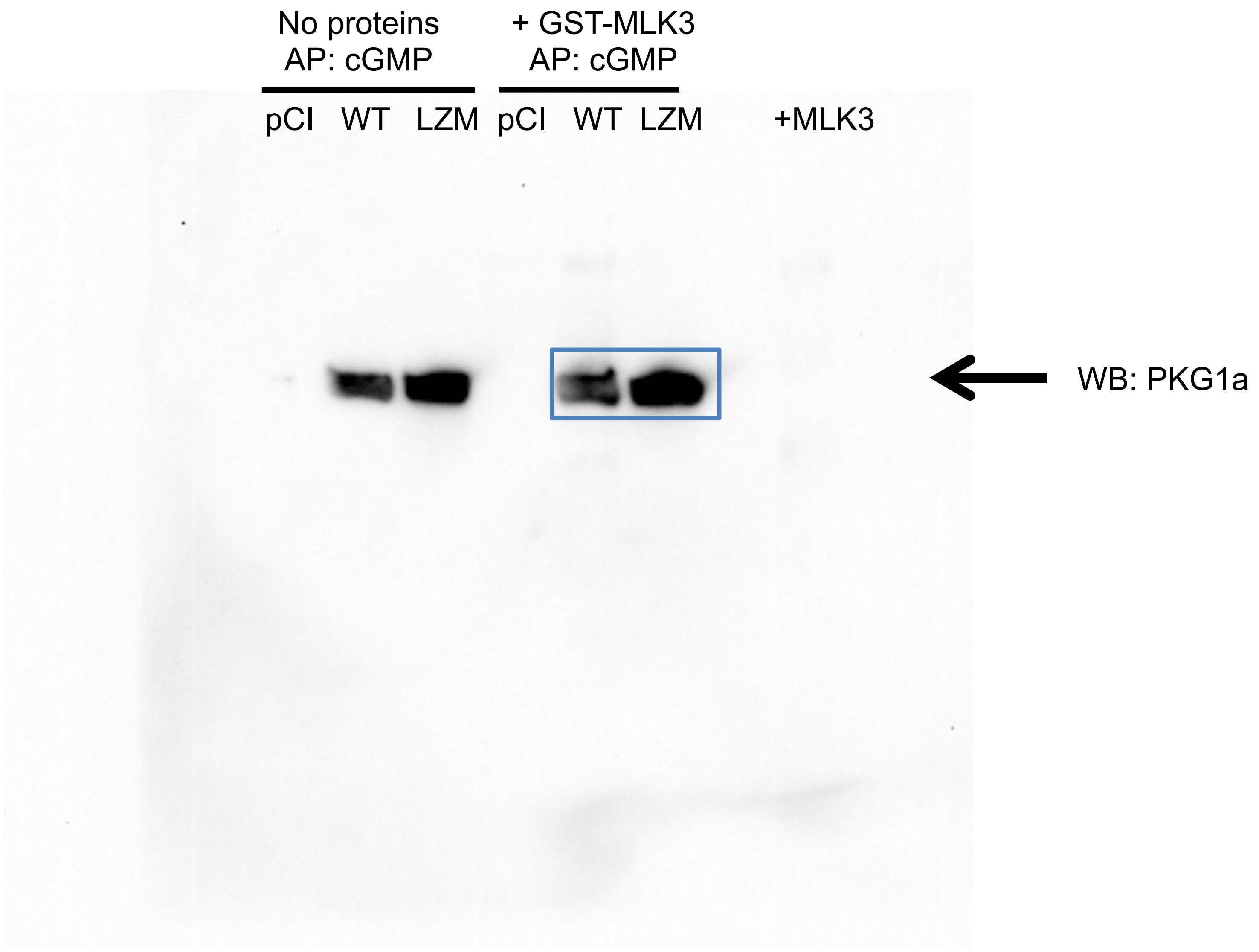

Cropped final Figure 2D

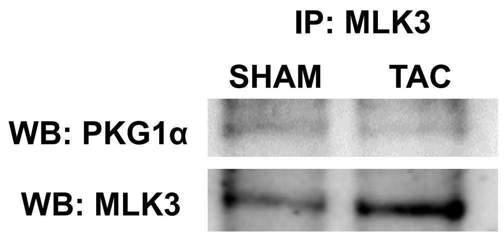

In this experiment we immunopreciptated MLK3 as described for Figure 2A, in LV lysates from wild type mice subjected to sham or to TAC surgery.

Full unedited gel for Figure 2D: Immunoblot MLK3

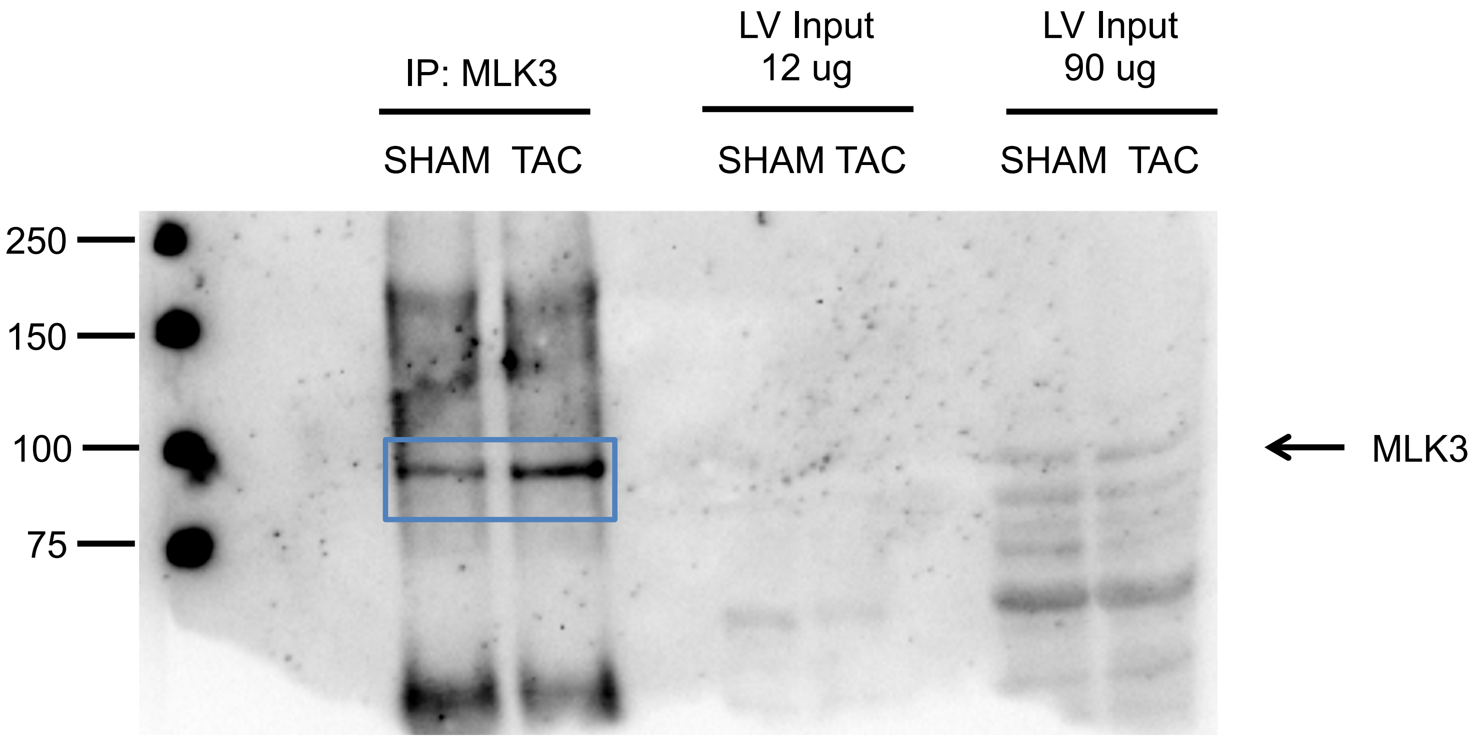

Full unedited gel for Figure 2D: Immunoblot PKGIα

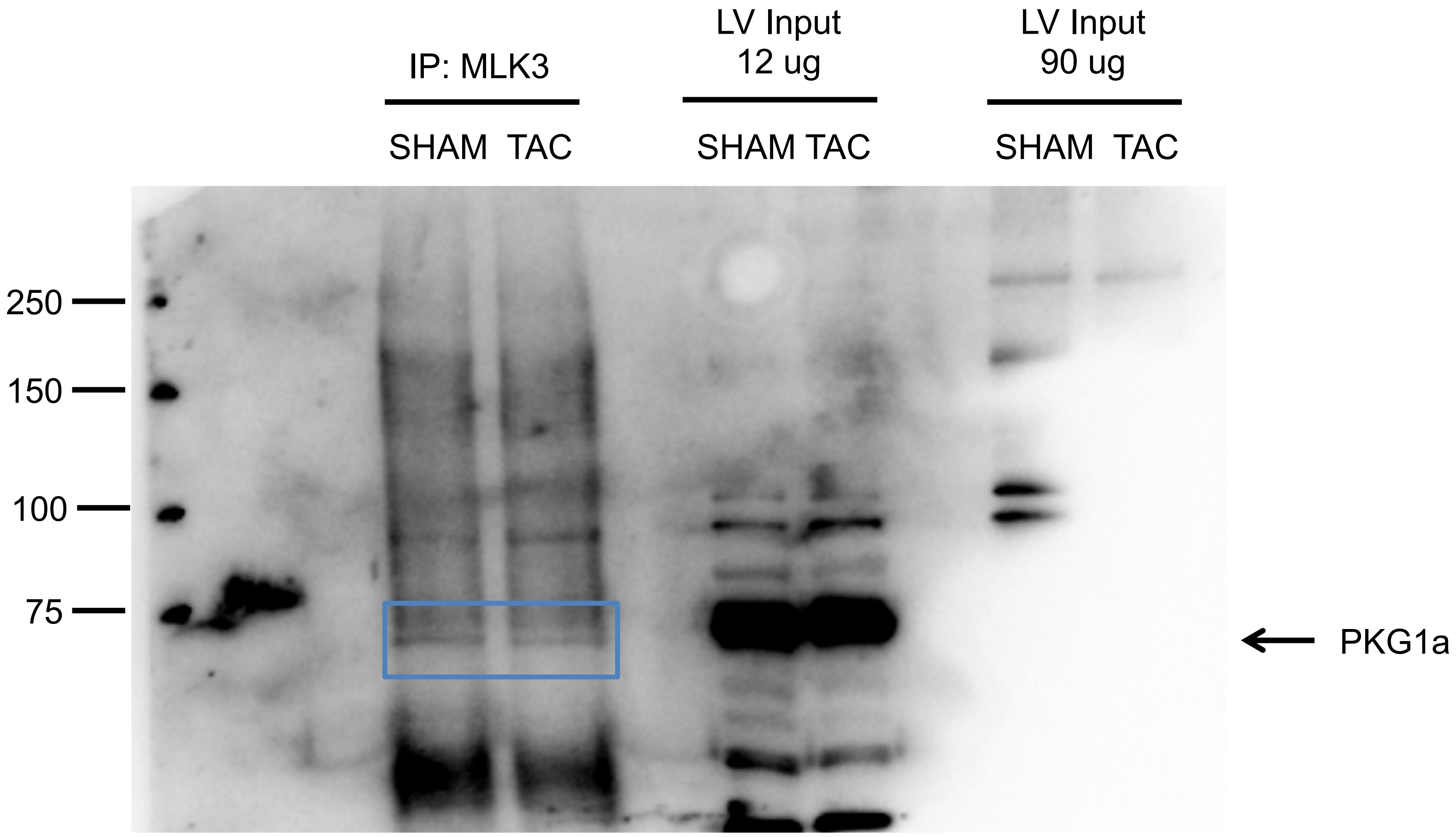

Cropped final Figure 2F

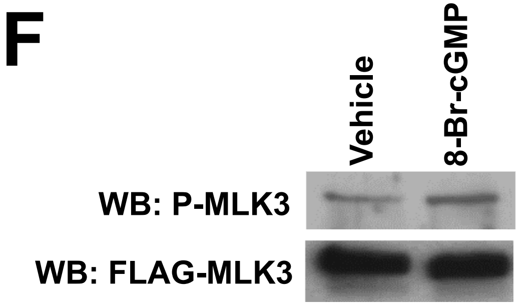

In this experiment only lanes 1 and 2 were used for the final image (lanes 3 and 4 are from an experiment with different conditions). Lane 5 is input control from FLAG-MLK3 transfected cells; Lane 6 is MLK3 positive control.

Full unedited gel for Figure 2F: Immunoblot phospho-MLK3

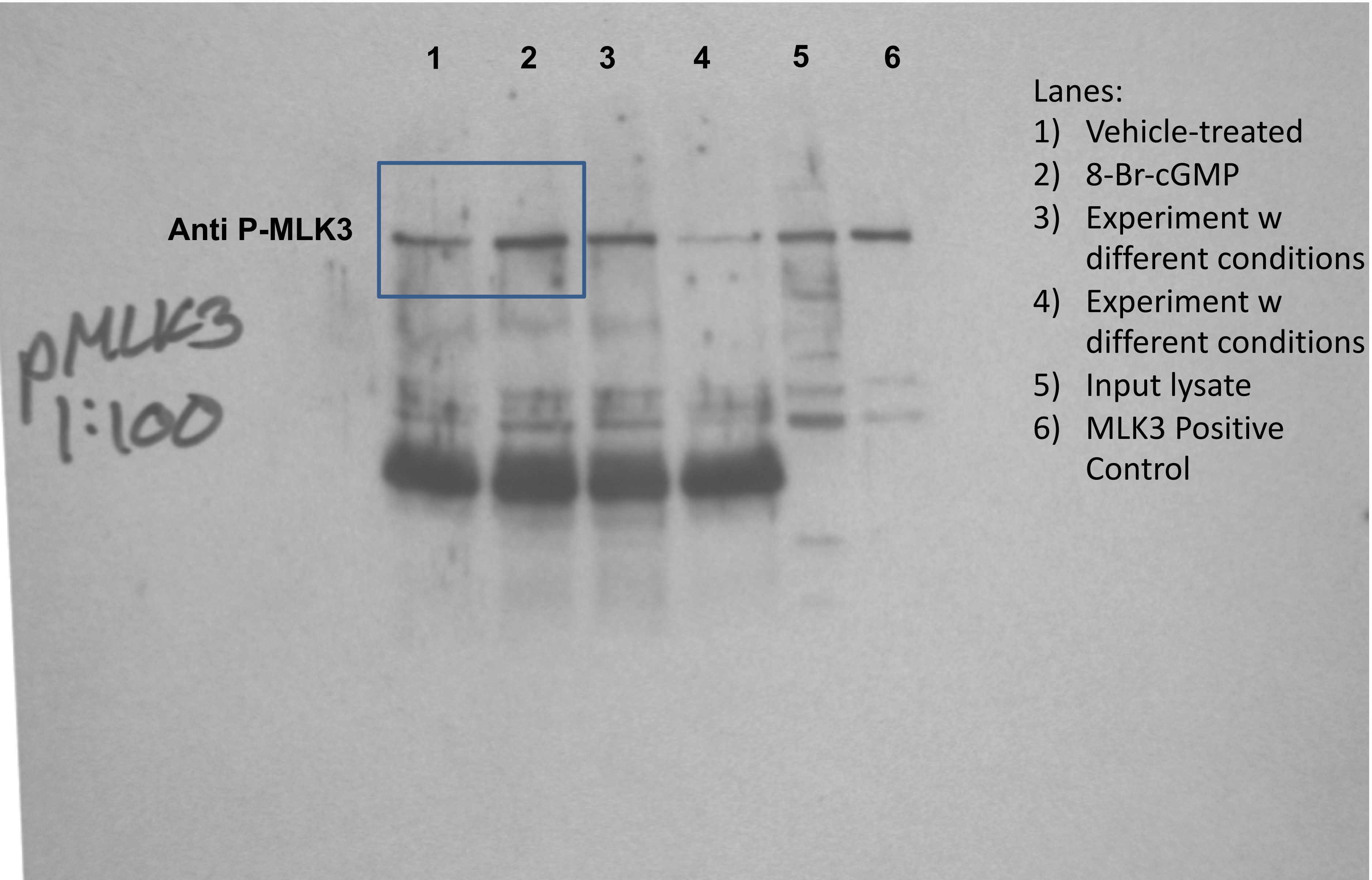

Full unedited gel for Figure 2F: Immunoblot FLAG

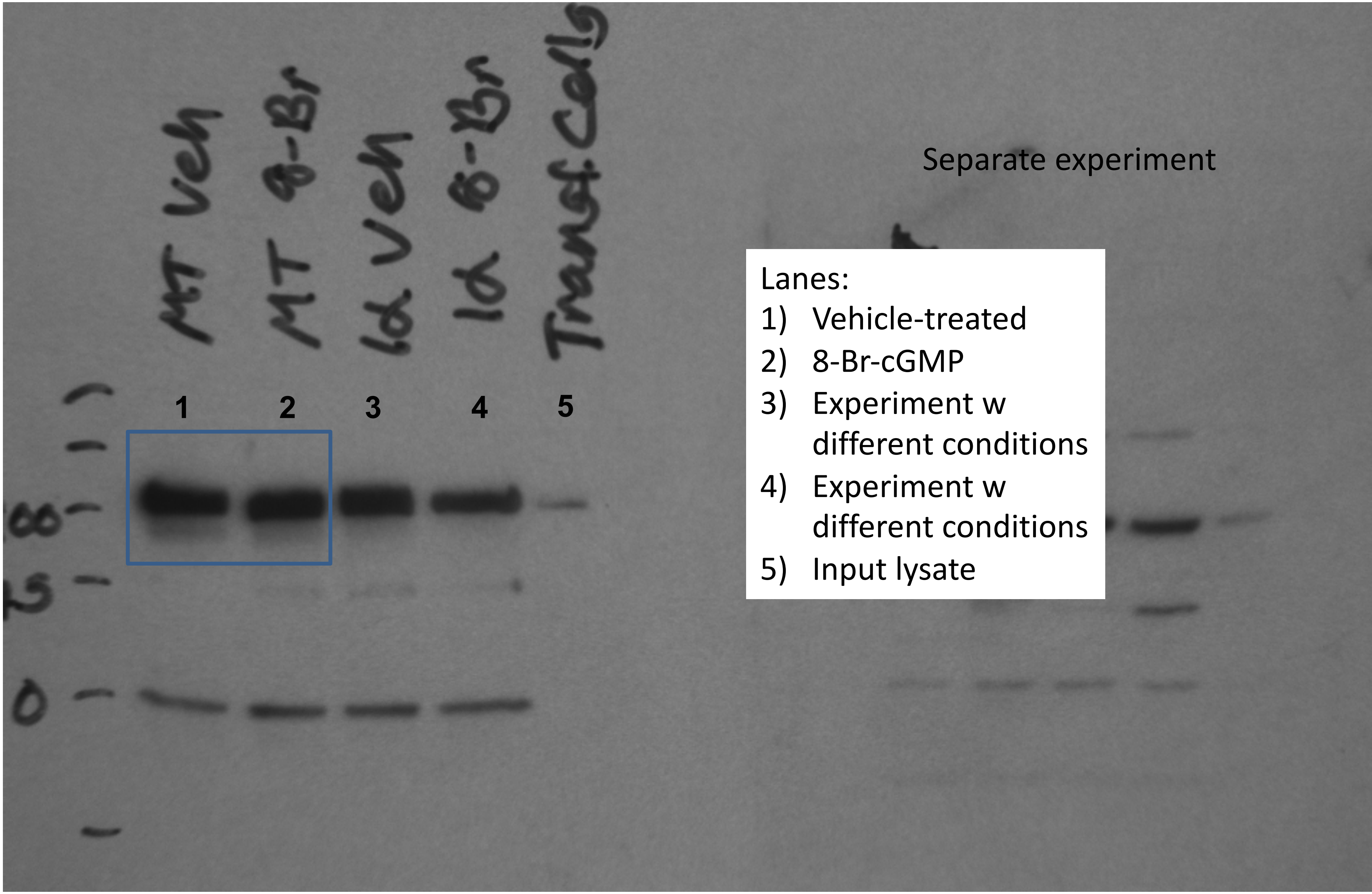

Full unedited gel for Figure 2F: Immunoblot FLAG

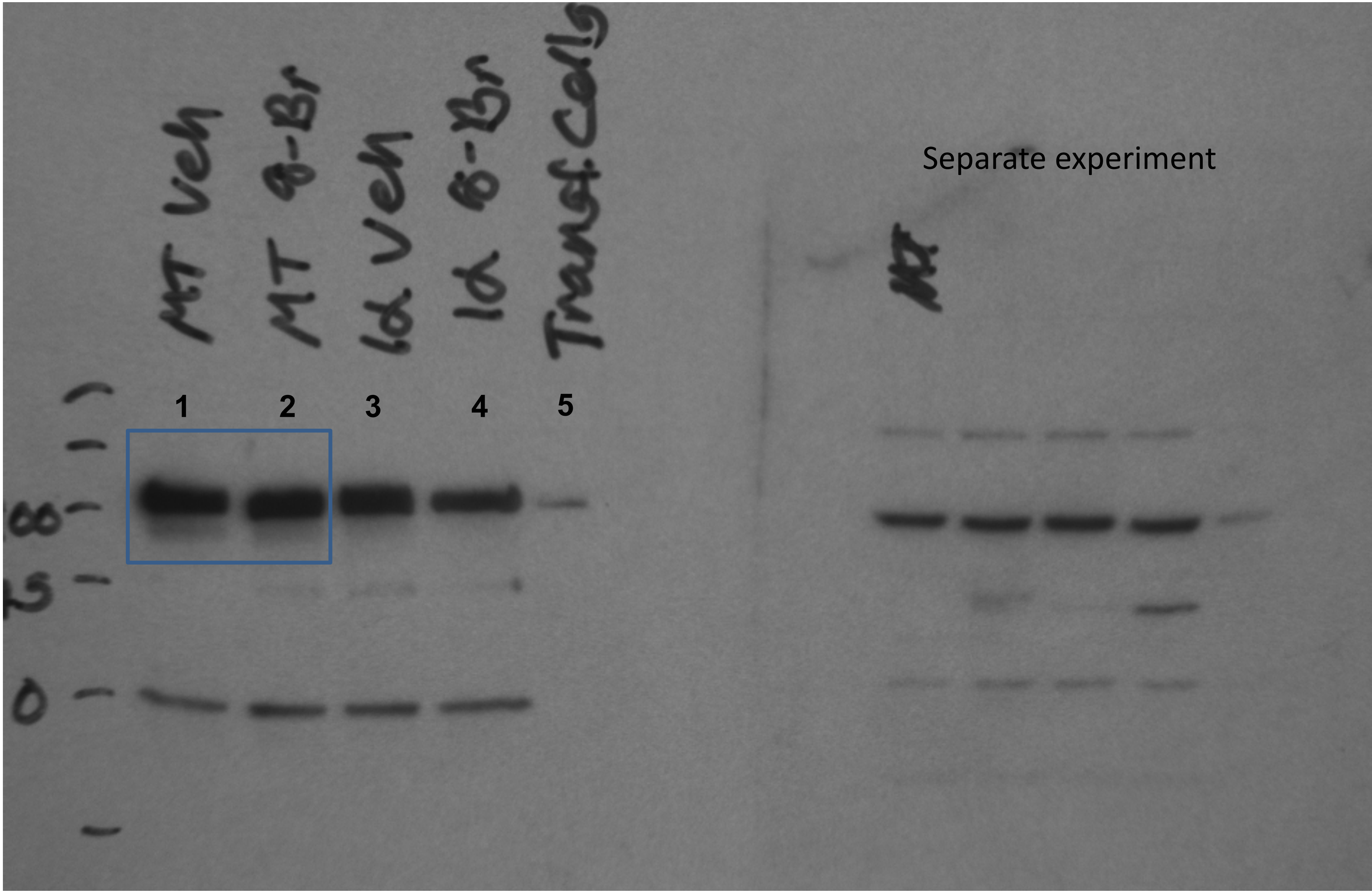

Cropped final Figure 2G

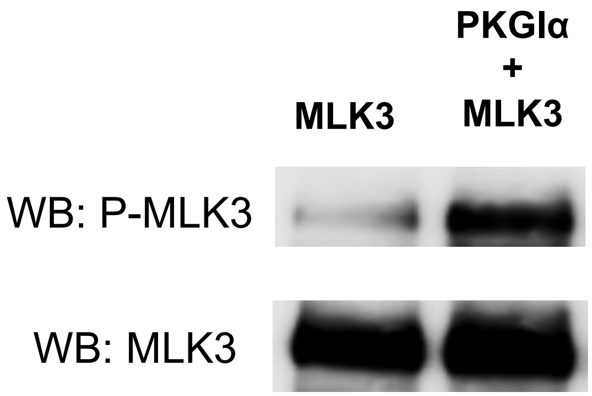

In this experiment only lanes 1 and 2 were used for the final image (lanes 3 and 4 are from an experiment with different conditions.

Full unedited gel for Figure 2G: P-MLK3 Immunoblot

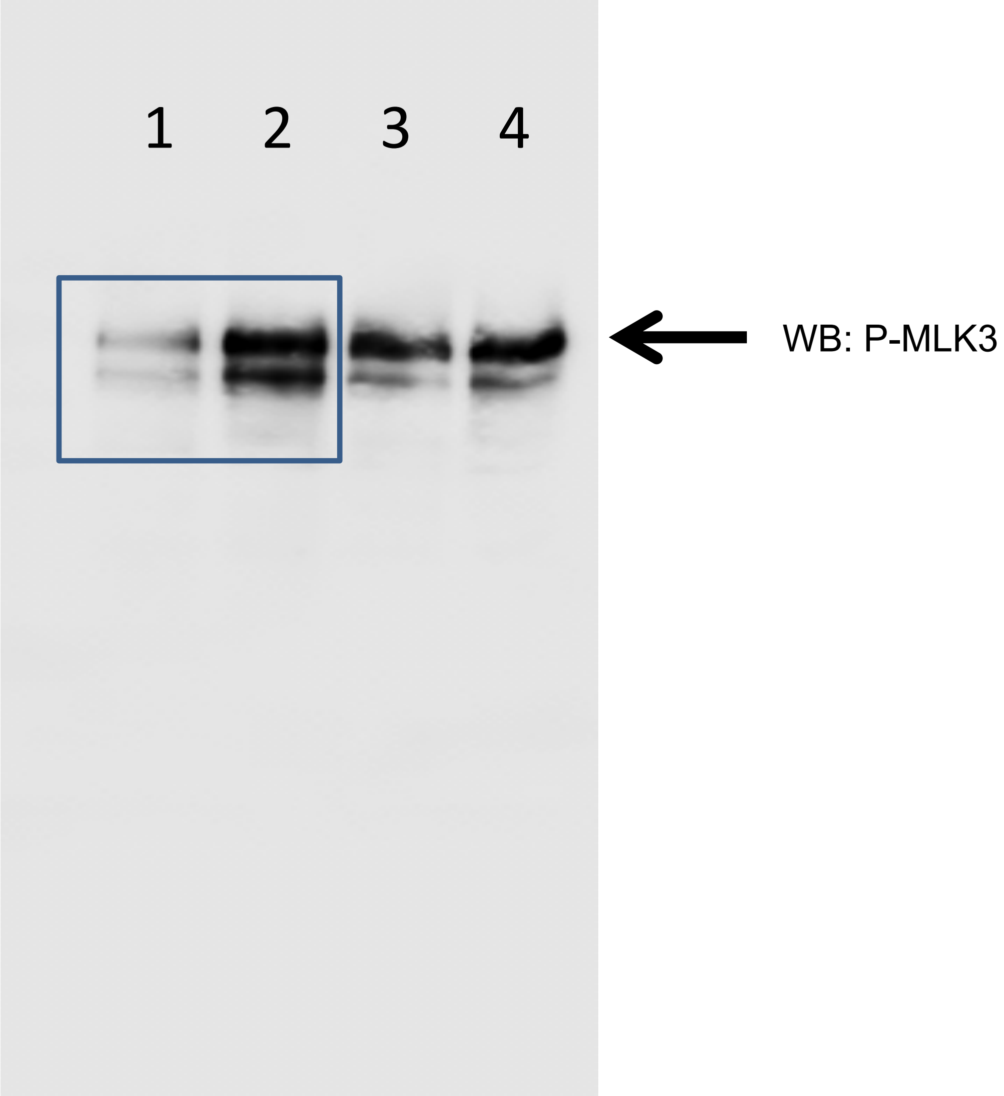

Full unedited gel for Figure 2G: MLK3 Immunoblot

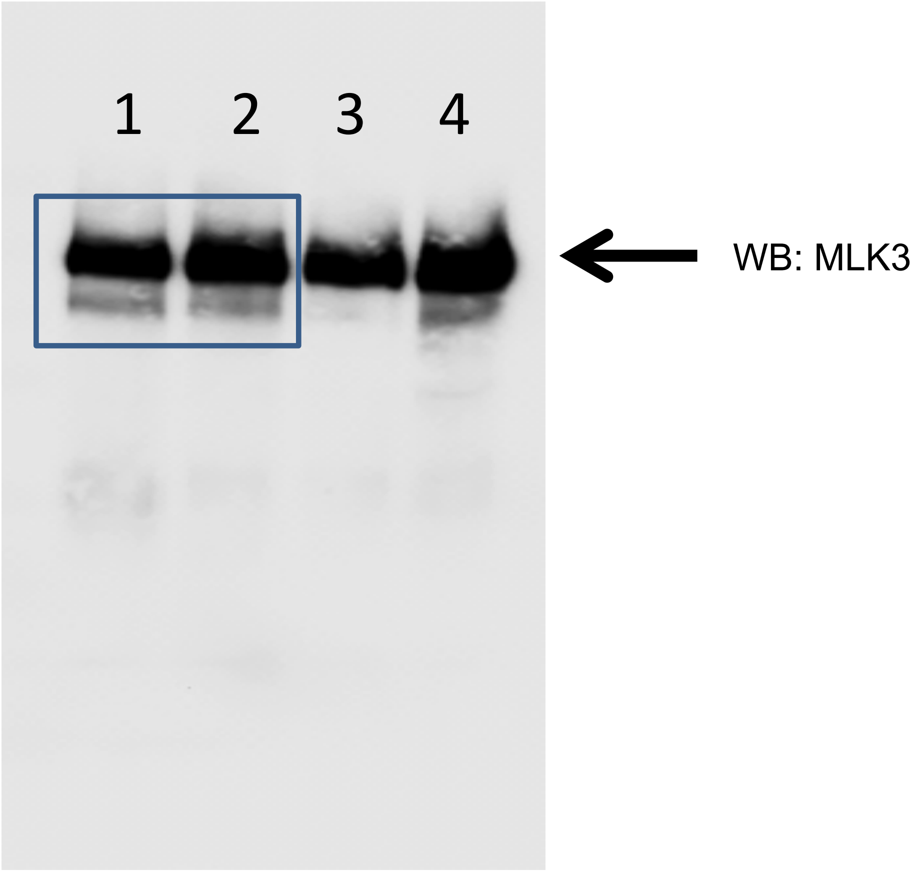

Supplement: Supplemental data [file jciinsight-6-149075-s010.pdf]
